# Modulation of DNA damage tolerance in *Escherichia coli recG* and *ruv* strains by mutations affecting PriB, the ribosome and RNA polymerase

**DOI:** 10.1111/mmi.12010

**Published:** 2012-09-07

**Authors:** Akeel A Mahdi, Geoffrey S Briggs, Robert G Lloyd

**Affiliations:** Centre for Genetics and Genomics, University of Nottingham, Queen's Medical CentreNottingham, NG7 2UH, UK

## Abstract

RecG is a DNA translocase that helps to maintain genomic integrity. Initial studies suggested a role in promoting recombination, a possibility consistent with synergism between *recG* and *ruv* null alleles and reinforced when the protein was shown to unwind Holliday junctions. In this article we describe novel suppressors of *recG* and show that the pathology seen without RecG is suppressed on reducing or eliminating PriB, a component of the PriA system for replisome assembly and replication restart. Suppression is conditional, depending on additional mutations that modify ribosomal subunit S6 or one of three subunits of RNA polymerase. The latter suppress phenotypes associated with deletion of *priB*, enabling the deletion to suppress *recG*. They include alleles likely to disrupt interactions with transcription anti-terminator, NusA. Deleting *priB* has a different effect in *ruv* strains. It provokes abortive recombination and compromises DNA repair in a manner consistent with PriB being required to limit exposure of recombinogenic ssDNA. This synergism is reduced by the RNA polymerase mutations identified. Taken together, the results reveal that RecG curbs a potentially negative effect of proteins that direct replication fork assembly at sites removed from the normal origin, a facility needed to resolve conflicts between replication and transcription.

## Introduction

The assembly of replication fork complexes at sites removed from the normal chromosomal origin plays a vital role in maintaining the integrity of the bacterial genome and in securing its duplication (Gabbai and Marians, 2010). In *Escherichia coli*, it relies on the PriA and PriC proteins to load the DnaB replicative helicase. Transfer of DnaB from a complex with DnaC to what becomes the template for lagging strand synthesis is a key step in fork assembly. Once loaded, DnaB recruits DnaG primase and PolIII holoenzymes, thus establishing a fully fledged fork complex, or replisome (Tougu *et al*., 1994; Kim *et al*., 1996a,b). Promiscuous loading of DnaB is prevented by prior binding of SSB protein to any exposed ssDNA (LeBowitz and McMacken, 1986). DnaA protein overcomes this barrier at *oriC* by opening the DNA in a sequence directed manner that excludes SSB (Messer, 2002). PriA and PriC achieve the same end, but in a sequence-independent manner at branched DNA structures.

The PriA system relies on PriA itself plus PriB and DnaT (Sandler and Marians, 2000; Gabbai and Marians, 2010). PriA is a DNA helicase with a 3′–5′ polarity of strand translocation. It has a strong affinity for three-strand junctions, enabling it to target a D-loop intermediate in recombination, or a fork structure, with high specificity (McGlynn *et al*., 1997; Nurse *et al*., 1999). PriB is related to SSB and binds with high affinity to ssDNA. It stabilizes a PriA–DNA complex, stimulates PriA helicase activity and facilitates binding of DnaT. The tripartite PriA–PriB–DnaT complex enables DnaB loading, thus nucleating replisome assembly (Cadman *et al*., 2005; Lopper *et al*., 2007; Gabbai and Marians, 2010). The PriC system appears to be directed at stalled forks, especially forks with a gap between the branch point and the 3′ leading strand hydroxyl (Heller and Marians, 2005). As with the PriA system, PriC facilitates DnaB loading in the presence of SSB. It can do so *in vitro* without the aid of other proteins (Heller and Marians, 2005), but may require the 3′–5′ helicase activity of either Rep or PriA to do so efficiently *in vivo* (Sandler, 2000; Mahdi *et al*., 2006; Gabbai and Marians, 2010).

Null mutations in *priA* reduce cell viability, compromise recombination and DNA repair, and block DnaA-independent, stable DNA replication (SDR). This pleiotropic phenotype is suppressed by missense mutations in *dnaC* (Sandler *et al*., 1996; 1999; Gregg *et al*., 2002). In the case of *dnaC810*, the altered DnaC protein overcomes the SSB barrier to load DnaB without the aid of PriA (Liu *et al*., 1999). A partial deletion of DnaT behaves much like a *priA* null (McCool *et al*., 2004). Surprisingly, a strain deleted for *priB* shows little loss of viability and is reasonably proficient in recombination and DNA repair. The same is true of a strain deleted for *priC*. However, a strain deleted for both *priB* and *priC* is barely viable (Sandler, 2000). Viability is improved by *dnaC809*, which encodes the same amino acid substitution as *dnaC810* (Sandler *et al*., 1996), and is restored to almost wild-type levels by *dnaC809,820*, which encodes an additional substitution (Sandler *et al*., 1999). On the basis of these and other observations demonstrating that *priA priC* and *priA rep* double mutants are inviable, Sandler (2000) concluded that there is cross-talk between the PriA and PriC systems, and proposed the existence of PriA–PriB, PriA–PriC and PriC–Rep pathways.

Although these pathways have evolved to promote cell survival, they establish a potential for replication to initiate when doing so offers no obvious advantage and might even be detrimental. Indeed, two proteins appear capable of curbing such activity, namely RNase HI and RecG. They reduce spurious initiations at R-loops, either by digesting the invading RNA strand or by unwinding the structure respectively (Horiuchi *et al*., 1984; Ogawa *et al*., 1984; Vincent *et al*., 1996; Fukuoh *et al*., 1997). Loss of either protein is associated with a substantial increase in DnaA-independent DNA synthesis. The loss of both is lethal (von Meyenburg *et al*., 1987; Asai and Kogoma, 1994a,b; Masai *et al*., 1994; Hong *et al*., 1995; Rudolph *et al*., 2009a,b).

Many features of the *recG* null phenotype are suppressed by mutations (e.g. *priA300*, *srgA1*) that reduce or eliminate the helicase activity of PriA (Al-Deib *et al*., 1996; Jaktaji and Lloyd, 2003; Rudolph *et al*., 2009a; Zhang *et al*., 2010). Unlike a *priA* null allele, these mutations do not reduce viability and retain the ability to promote DNA repair and recombination (Kogoma *et al*., 1996; Sandler *et al*., 1996; Jaktaji and Lloyd, 2003). The *srgA1* allele of *priA* is especially informative. The mutant protein unwinds a three-way branched structure mimicking a replication fork. However, it has lost the ability to unwind a 3′ flap structure mimicking a fork with no leading strand at the branch point (Gregg *et al*., 2002), a structure RecG unwinds with high efficiency (McGlynn and Lloyd, 2001; Tanaka and Masai, 2006). This has led to the idea that 3′ flaps are generated accidentally during replication, but are eliminated *via* the combined actions of RecG and ssDNA exonucleases. Without RecG to unwind the structure, PriA is more likely to target the flap, thus triggering replisome assembly and re-replication of the already replicated DNA, with pathological consequences (Rudolph *et al*., 2009b; 2010a).

In this work, we describe how reducing or abolishing PriB can also lead to suppression of the *recG* null phenotype. However, the suppression requires additional mutations that alter 30S ribosomal subunit S6, or one of three major subunits of RNA polymerase, namely RpoA, RpoB or RpoC. These RNA polymerase mutations suppress a negative feature of the deletion *priB* phenotype that masks the ability to suppress *recG*. They also reduce a synergism between *priB* and *ruv* null alleles that we attribute to abortive recombination provoked by the exposure of ssDNA. We conclude that RecG is needed to curb a potential danger of replisome assembly directed at sites removed from *oriC* by the PriA system, a facility required to resolve conflicts between DNA replication and transcription.

## Results

**Fig. 1:**
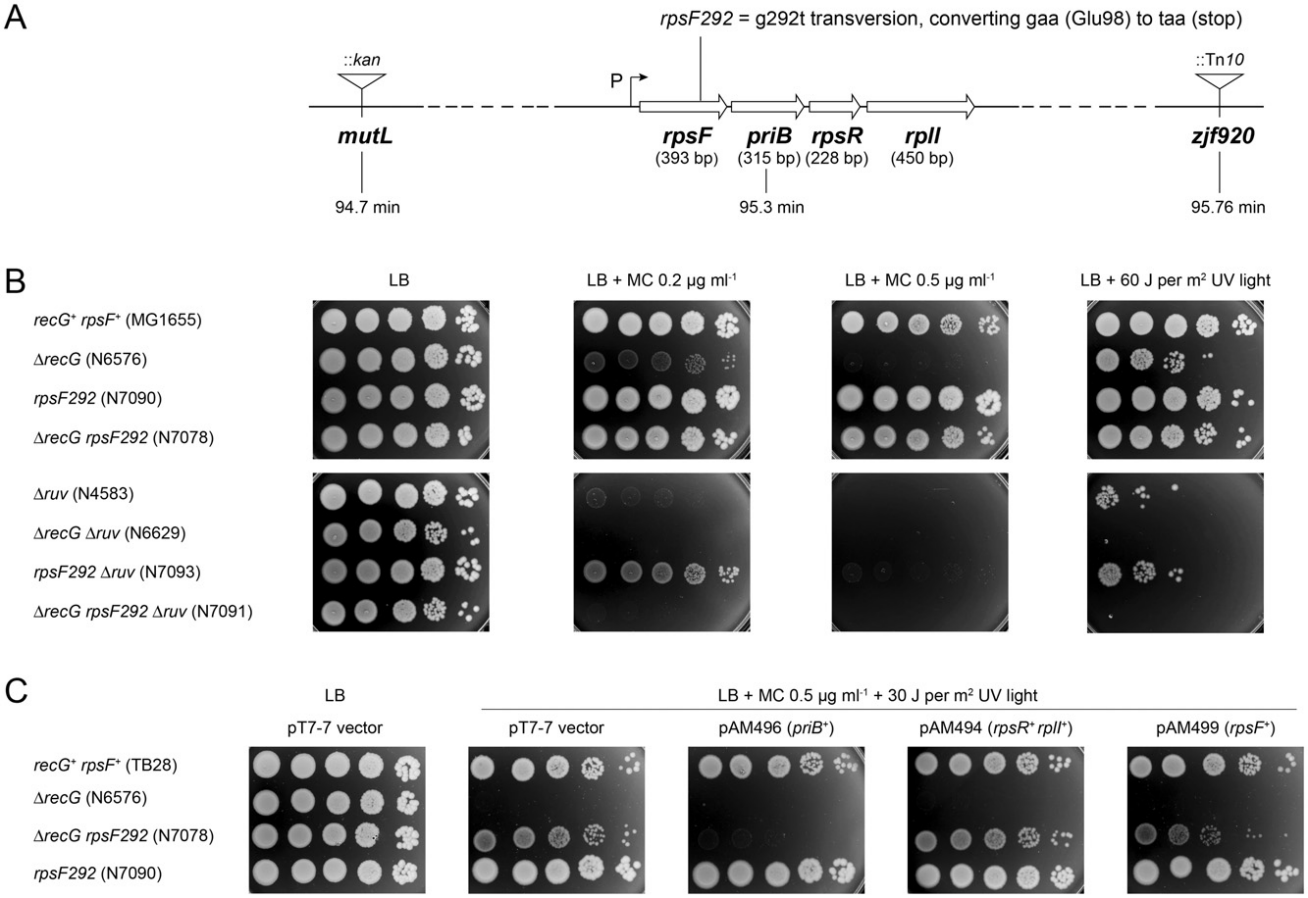
Suppression of *recG* by *rpsF292*. A. Chromosomal location of *rpsF* and of downstream genes expressed from the same promoter (P). The position of the *rpsF292* mutation and flanking markers exploited is also shown. B. Effect of *rpsF292* on the sensitivity of *recG* and *ruv* strains to mitomycin C and UV light. The strains examined are identified by genotype, followed in each case by the strain number in parentheses. C. Expression of wild-type RpsF or PriB *in trans* reduces *rpsF292* suppression of *recG*. Except for the presence of the indicated plasmid, the strains examined are identified by genotype, followed in each case by the strain number in parentheses.

**Fig. 2:**
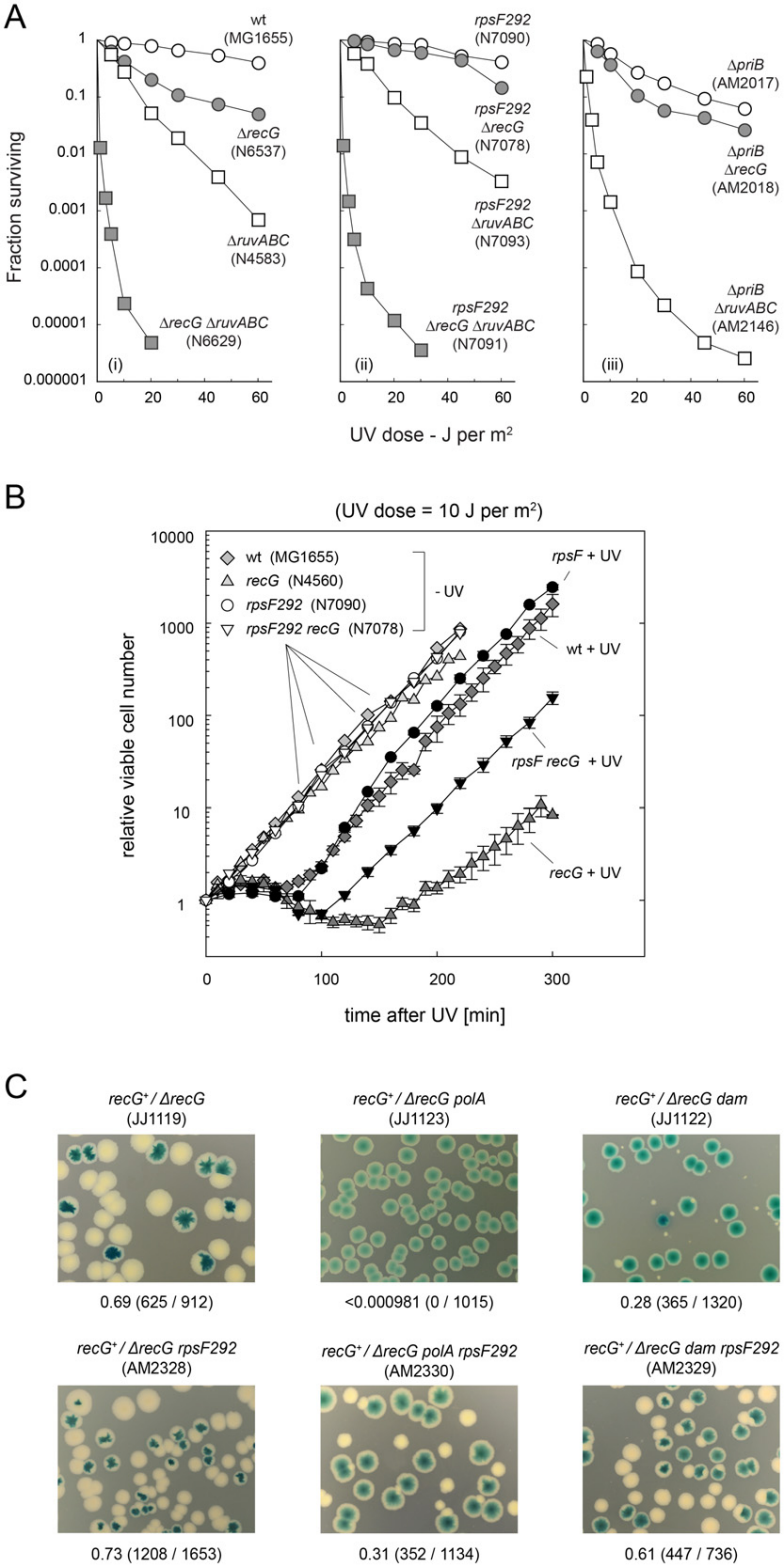
Effect of *rpsF292* and Δ*priB* on the *recG* and *ruv* mutant phenotypes. A. Sensitivity to UV light. The strains examined are identified by genotype, with the strain number in parentheses *below* the genotype. B. Cell replication following UV irradiation. Strain genotypes are as identified, with strain numbers in parentheses. Data are means (± SE) of three independent experiments for irradiated and two for unirradiated cells. Data for MG1655 (wt) and its *recG* derivative, N4560, are reproduced for comparison from Rudolph *et al*. (2007b) and Rudolph *et al*. (2009a) respectively. C. Synthetic lethality assays showing how *rpsF292* overcomes the inviability of *recG polA* and *recG dam* cells. The plate assay exploited here and in subsequent figures is described in detail in *Experimental procedures*. The relevant genotype of the construct used is shown *above* the section of the plate photograph displayed. In each case the relevant plasmid genotype/relevant chromosome genotype (e.g. *recG*^*+*^*/*Δ*recG*) is indicated, along with the strain number in parentheses. The fraction of white (Lac^−^) colonies is shown *below* with the number of white colonies/total colonies analysed in parentheses. White colonies arise from cells that lost the plasmid before plating whereas blue (Lac^+^) colonies or blue/white, sectored colonies arise from those that retained the plasmid.

Recent studies exploiting *priA* and *ssb* suppressors of the *recG* null phenotype revealed how RecG protein might limit pathological events that disrupt the normal course of chromosome duplication (Rudolph *et al*., 2009a,b; 2010a,b; Zhang *et al*., 2010). In a new screen of Δ*recG* derivatives selected for increased resistance to mitomycin C we isolated a novel clone that proved wild type for both *priA* and *ssb*. It carries instead a mutation in the *rpsF* gene encoding 30S ribosomal subunit S6 (Supplementary results). The G to T transversion identified and labelled *rpsF292* converts the GAA codon for Glu98 to a TAA stop codon (Fig. 1A). This nonsense allele confers no obvious phenotype on its own, but is an effective and general suppressor of *recG*. Thus, it restores resistance to mitomycin C (Fig. 1B), alleviates the slight sensitivity to UV light (Fig. 1B and 2A, panels i and ii), and reduces the extended delay in replication of those cells surviving irradiation (Fig. 2B). It also overcomes the requirement for both Pol I and Dam proteins to maintain robust growth on LB agar (Fig. 2C), and improves the recovery of recombinants in conjugational and transductional crosses (Table 1). Its ability to do so depends on the presence of the RuvABC Holliday junction resolvase (Fig. 1B and 2A, panels i and ii; Table 1).

**Table 1:** Effect of *rpsF292* on conjugational DNA transfer and recombination

			Relative number of transconjugants or P1 transductants^b^				
			
			x KL548	Hfr GY2200		Hfr KL226	
				
Strain number	Relevant genotype	Relative viability^a^	(F′ Pro^+^)	(λ)^c^	(Thr^+^Leu^+^)	(Pro^+^)	P1 transductants (Leu^+^)
AB1157	*rps^+^ rec^+^ ruv^+^*	1.0	1.0	1.0	1.0	1.0	1.0
N7962	*rpsF292*	0.93	1.18	1.22	0.99	1.17	0.82
AM2123	Δ*recG*	0.82	0.7	0.89	0.35	0.25	0.14
N7985	*rpsF292* Δ*recG*	0.78	1.29	1.27	0.73	0.91	0.43
N4454	Δ*ruvABC*	0.62	0.62	0.84	0.42	0.43	0.21
N7986	*rpsF292* Δ*ruvABC*	0.60	1.13	1.15	0.51	0.52	0.15
AM2124	Δ*recG* Δ*ruvABC*	0.28	0.21	0.67	0.0018	0.0014	0.0011
N7987	*rpsF292* Δ*recG* Δ*ruvABC*	0.23	0.28	1.06	0.0024	0.0020	0.0048

a. Values for cell viability are based on the recipient cultures used in conjugational crosses. Those based on cultures of the same recipients used in P1 transductions are shown in Table S2. Although the culture conditions are not the same, the two estimates are generally very close.

b. Mating was for 30 (KL548), 40 (KL226) or 60 (GY2200) min and the transconjugant class selected is indicated. The phage P1 donor was strain W3110. Values for wild-type control strain AB1157 are set at 1. The actual mean values ± SE are shown in Table S2. Mutant strains were tested in parallel with AB1157 and the values shown are mean yields relative to AB1157 in each of three or more experiments. Numbers of experiments and standard errors are provided in Table S2.

c. λ plaques arise from zygotic induction of the λ prophage transferred by the Hfr.

The stop codon introduced by *rpsF292* would be expected to eliminate the final 35 amino acids from the C-terminus of RpsF, the final two glutamic acids of which are needed for post-translational addition of a further four glutamates (Reeh and Pedersen, 1979; Kang *et al*., 1989). It might also cause premature termination of transcription and thus reduce expression of the downstream genes transcribed from the *rpsF* promoter. Significantly, these genes include *priB*, which is associated with the PriA system of replication restart. Previous studies revealed that mutations affecting the helicase activity of PriA suppress the sensitivity of *recG* cells to mitomycin-C (Al-Deib *et al*., 1996; Jaktaji and Lloyd, 2003). To determine which of these effects of *rpsF292* might account for the suppression of *recG*, we introduced plasmids encoding the downstream genes into an *rpsF292* Δ*recG* double mutant. A *priB^+^* construct makes the strain almost as sensitive to a combination of mitomycin C and UV light as a Δ*recG* single mutant (Fig. 1C). In contrast, a plasmid encoding *rpsR^+^* and *rplI^+^* behaves like the vector. Thus it seems that reduced expression of PriB might be a substantial factor. However, a plasmid encoding *rpsF^+^* also reduces resistance (Fig. 1C). The effect is not as great as seen with the *priB^+^* plasmid, but the fact that there is any reduction in sensitivity at all does suggest that the truncation of RpsF contributes to the strength of the suppression.

### Δ*rpsF* and Δ*priB* are weak suppressors of the *recG* mutant phenotype

**Fig. 3:**
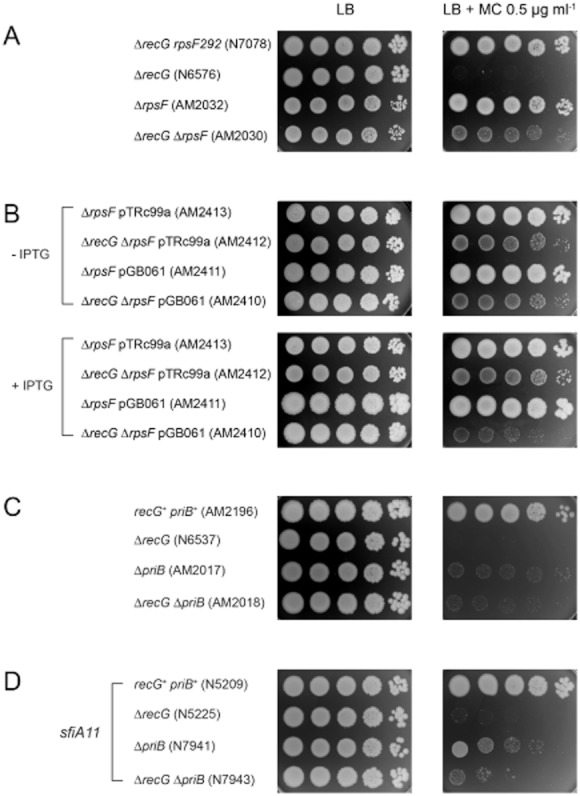
Effect of RpsF, PriB and SfiA depletion on sensitivity to mitomycin C. A, C and D. Effect of *rpsF*, *priB* and *sfiA* null alleles, respectively, in the presence and absence of RecG. B. Expression of *rpsF*^+^ *in trans* improves growth of Δ*rpsF* cells and reduces the suppression of Δ*recG*. The strains examined are identified by genotype, followed in each case by the strain number in parentheses.

We made in-frame deletions of *rpsF* and *priB* to examine directly whether loss of either would suppress *recG*. Neither is essential for growth (Sandler *et al*., 1999; Bubunenko *et al*., 2007). The Δ*rpsF* allele clearly alleviates sensitivity to mitomycin C, although it is not as effective as *rpsF292* (Fig. 3A). The resistance conferred is reversed by expressing *rpsF^+^* from a plasmid (Fig. 3B). Given any polar effect of the *rpsF* deletion on downstream genes would persist in the presence of the *rpsF^+^* plasmid, these data support the notion that inactivation of *rpsF* contributes substantially to the observed suppression of *recG*.

The Δ*priB::dhfr* allele we made confers slight sensitivity to UV light and moderate sensitivity to mitomycin C (Fig. 2A, panel iii; Fig. 3C). Another deletion, Δ*priB202* (Sandler *et al*., 1999), made without a resistance tag confers similar sensitivity to mitomycin C (data not shown). Neither allele is able to confer wild-type resistance to mitomycin C on a *recG* strain (Figs 3C and S2A). The *recG priB* double-deletion strain also remains slightly sensitive to UV light (Fig. 2A, panel iii). However, a side-by-side comparison reveals that a *priB* single mutant is not quite as sensitive to mitomycin C as a *recG* strain, and that a *recG priB* double mutant behaves like a *priB* strain (Fig. 3C and data not shown), indicating that there is some weak suppression of *recG*.

We investigated whether the sensitivity of a *priB* strain to mitomycin C might be due to increased expression of the SOS-induced division inhibitor encoded by *sfiA* (*sulA*). Previous studies had shown that *sfiA* inactivation enhances the viability of *priA* null cells (Nurse *et al*., 1991). We observed that it also improves the growth of both *priB* and *priB recG* strains in the presence of mitomycin C. However, the improvement is quite modest (Fig. 3D). There is no improvement with a *recG* strain. Taken together, these observations confirm that the *recG* phenotype is partially suppressed by the elimination of either RpsF or PriB. They are consistent with the notion that the strong suppression observed with *rpsF292* is due to the combined effect of mutating RpsF and reducing the expression of PriB.

### RNA polymerase mutations suppress Δ*priB* and enable Δ*priB* to suppress Δ*recG*

Despite both Δ*recG* and Δ*priB* conferring sensitivity to mitomycin C, cultures of the double mutant readily accumulate resistant derivatives, suggesting that a single additional mutation might suffice to suppress sensitivity. We isolated 18 resistant clones of the *recG priB* strain AM2055 (Fig. S1), and established by DNA sequencing and genetic reconstruction that mutation of a single gene is responsible for the alleviation of sensitivity in at least 14 of these cases.

In no case was the suppressor an allele of *priA*. Instead, the mutations identified were located to genes encoding one of three major subunits of RNA polymerase. Several were found in *rpoA* and *rpoB*, and one in *rpoC*, with some alleles appearing more than once (Table 2). The *rpoA[P293L]* allele confers a requirement for methionine or cysteine for growth. The same requirement was previously associated with a K271E substitution (Thomas and Glass, 1991). It enabled us to identify *rpoA[P293L]* repeatedly in a further screen of Δ*recG* Δ*priB* strains selected for resistance to mitomycin C (Table S1 and strains not listed). The same screen also identified two independent *rpoA* isolates encoding a K298N substitution.

**Table 2:** Properties of *rpo* suppressors of Δ*priB* and Δ*priB* Δ*recG*

Suppressorisolate^a^	Gene affected	DNA sequence change(s)^b^	Allele designation	RNAP feature affected	Rifampicin resistance^c^	Stringent phenotype^d^	rpo^*^ activity^e^
AM2064/2066	*rpoA*	CCT (Pro293) to CTT (Leu)	*rpoA[P293L]*	Alpha C-terminal domain	< 5	ND	Weak negative
AM2072/2075							
AM2067	rpoA	CTG (Leu253) to CGG (Arg)	*rpoA[L253R]*	Alpha C-terminal domain	< 5	ND	Weak negative
AM2074	*rpoA*	GAA (Glu273) to GAT (Asp)	*rpoA[E273D]*	Alpha C-terminal domain	< 5	None	Neutral
AM2174	*rpoA*	AAA (Lys298) to AAT (Asn)	*rpoA[K298N]*	Alpha C-terminal domain	< 5	ND	Weak negative
AM2071	*rpoA*	TCA (Ser49) to ACA (Thr)	*rpoA[S49T,S309P]*	Alpha C-terminal domain (S309P)	< 5	ND	Negative
		TCC (Ser309) to CCC (Pro)					
AM2070	*rpoB*	CGT (Arg452) to CTT (Leu)	*rpoB[R452L]*	Non-transcribed ssDNA channel	10	Very weak	Weak negative
AM2073	*rpoB*	GGT (Gly1260) to GAT (Asp)	*rpoB[G1260D]*	RNA exit channel	10	Strong	Positive
AM2060/2069	*rpoB*	TCG (Ser1332) to TTG (Leu)	*rpoB[S1332L]*	RpoB:RpoC interface; RNA exit?	< 5	Strong	Weak positive
AM2063	*rpoB*	Δ(G1336-C1344)	*rpoB[*Δ*D446-L448]*	Point of template DNA re-annealing	5	Very weak	Positive
AM2059	*rpoC*	Δ(A643-T660)	*rpoC[*Δ*K215-R220]*	β′_B_ rudder in the DNA channel?	< 5	Strong	Negative

a. Except for AM2174, the suppressor isolates are derivatives of strain AM2055 (Δ*lacIZYA* Δ*recG*::*apra zjf920*::Tn*10* Δ*priB202*) selected for their resistance to mitomycin C. AM2064 and AM2066 came from the same culture of AM2055 and therefore may be siblings. AM2072 and AM2075 could also be siblings, but are independent of AM2064 and AM2066. AM2174 is a mitomycin C-resistant derivative of AM2167 (Δ*lacIZYA* Δ*recG*::*apra zjf920*::Tn*10* Δ*priB202 yheR*::*kan*). The *rpoA[P293L]* allele was also identified in two other independent isolates, namely AM2173 and AM2191 (Table S1).

b. As defined in parentheses by the amino acid substitution(s) or deletion.

c. Strains were tested for growth on LB agar supplemented with rifampicin to a final concentration of 5, 10, 15, 20 or 50 μg ml^−1^. The parent strains show no resistance to rifampicin at 5 μg ml^−1^. The maximum concentration of rifampicin allowing growth to single colonies is indicated.

d. As determined by the ability of the *rpo* allele to allow a *relA spoT* strain to grow on minimal agar, i.e. to confer prototrophy (Cashel *et al*., 1996).

e. As determined from the survival of a Δ*ruvABC* derivative irradiated with UV light at doses ranging from 5 to 60 J per m^2^ (McGlynn and Lloyd, 2000). Neutral: no effect; positive: improves survival; negative: reduces survival.

**Fig. 4:**
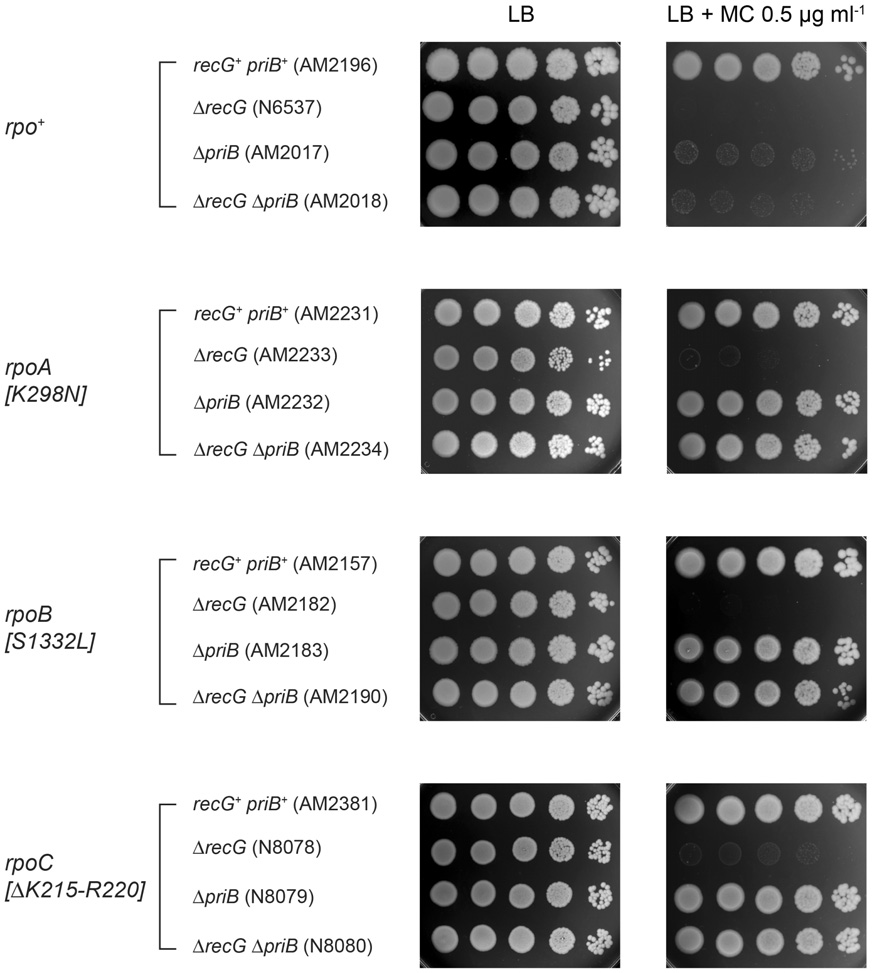
Effect of RNA polymerase mutations on sensitivity to DNA-damaging agents. Suppression of the sensitivity of *priB* and *priB recG* cells to mitomycin C by mutation of RpoA, RpoB or RpoC. The strains examined are identified by genotype, followed in each case by the strain number in parentheses.

We transferred the *rpo* alleles to wild-type strain MG1655 and examined the sensitivity to mitomycin C of the *rpo* single mutant constructs and of derivatives carrying Δ*recG*, Δ*priB* or both. The *priB* and *priB recG* derivatives all proved quite resistant, as did the *rpo* single mutants. However, the *recG* derivative remained sensitive in every case, although slightly increased resistance was observed in a few instances, notably with *rpoA[L253R], rpoA[E273D]* and *rpoB[*Δ*D446-L448]* (Figs 4 and S2). These data demonstrate that the *rpo* mutations are suppressors of Δ*priB* and when present enable Δ*priB* to strongly suppress Δ*recG*.

The *rpoB[G1260D]* allele was identified previously among a subclass of stringent RNAP mutations that improve survival of UV-irradiated strains lacking the RuvABC Holliday junction resolvase (McGlynn and Lloyd, 2000; Trautinger and Lloyd, 2002). We considered whether suppression of *priB* might be a general property of these so-called rpo* mutations (McGlynn and Lloyd, 2000). We tested rpoB*35, which encodes an H1244Q substitution in the β-subunit that appears to destabilize transcription elongation complexes (McGlynn and Lloyd, 2000; Trautinger *et al*., 2005). This allele clearly increases the resistance of a *priB* strain to mitomycin C, but has little or no effect on a *recG* strain unless *priB* is deleted (Fig. S2J). However, with the exception of *rpoB[G1260D]*, the *rpo* alleles identified here seem distinct from the rpo* class. Only one (*rpoB[R452L]*) confers the modest resistance to rifampicin characteristic of both rpoB*35 and *rpoB[G1260D]*, and only two (r*poB[S1332L]* and *rpoC[*Δ*K215-R220]*) confer a stringent phenotype (Table 2). The ability to affect the survival of UV-irradiated Δ*ruvABC* cells also varies. Again, apart from *rpoB[G1260D]*, which has a strong positive effect, only *rpoB[*Δ*D446-L448]* shows an ability to improve survival. Indeed, several have a substantial negative effect (Table 2; Fig. S3). No *rpoA* alleles were identified among the rpo* class of *ruv* suppressors described previously. It is also significant that the *rpoA* alleles identified here encode substitutions in RpoA that are unlikely to impinge on the DNA channel through RNA polymerase, a notable feature of the rpo* class (Trautinger and Lloyd, 2002). They appear instead to affect a C-terminal domain of the RpoA subunit that interacts with the transcription anti-terminator, NusA (Mah *et al*., 2000).

From these data it is clear that eliminating PriB has itself a significant negative effect on the ability of cells to withstand damage to their DNA. We probed Δ*priB* strains in more detail to see if we could shed light on how the absence of PriB is able nevertheless to mask the *recG* phenotype and explain why its ability to do so is conditional on some alteration of RNA polymerase. We focused initially on cells lacking the RuvABC resolvase since previous studies demonstrated that the *priA300* suppressor of *recG* has a negative effect on DNA repair in such cells (Jaktaji and Lloyd, 2003).

### The absence of PriB provokes recombination

**Fig. 5:**
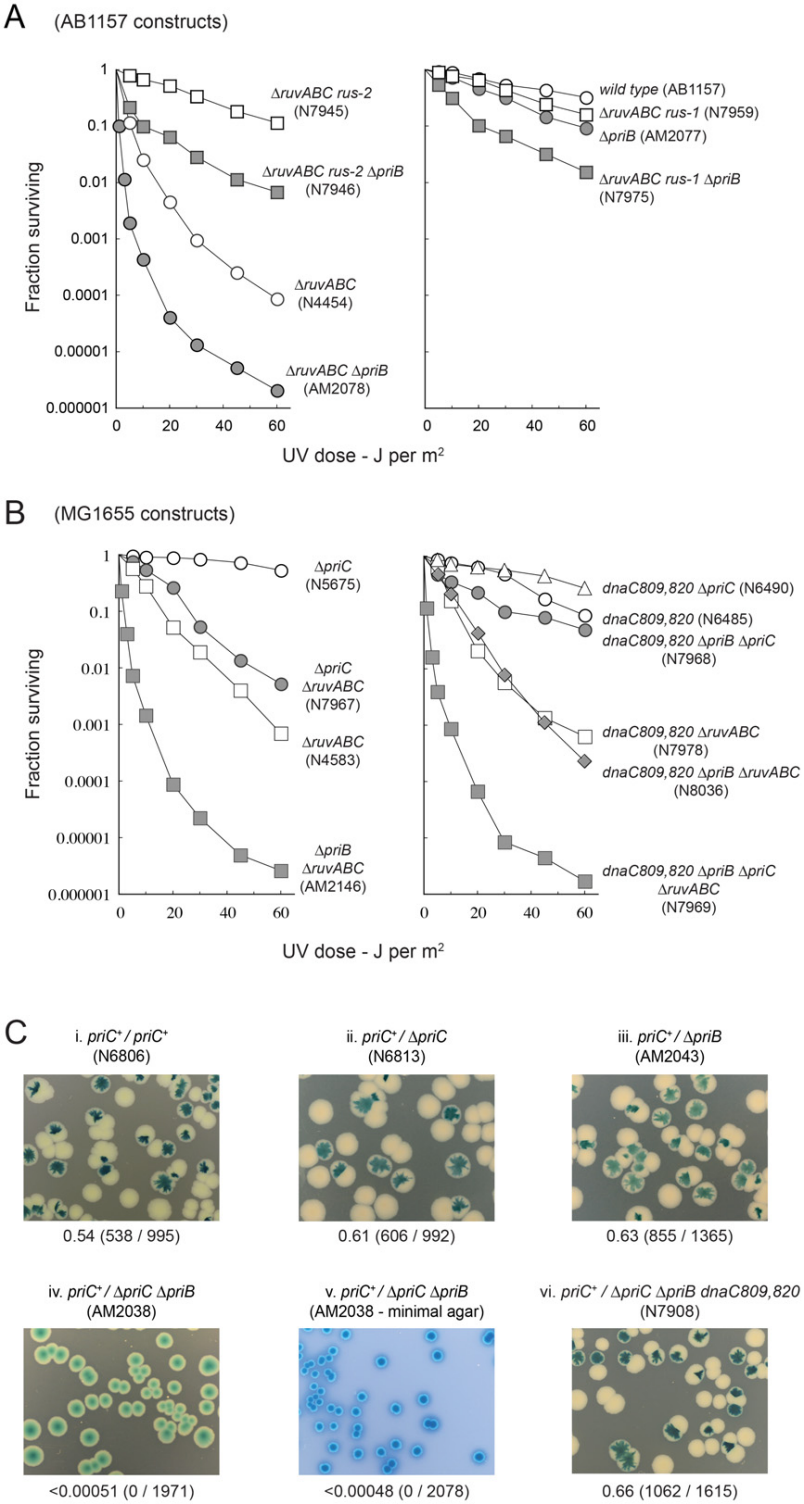
Suppression of the *priB* mutant phenotype. A and B. Suppression of the synergism between *priB* and *ruv* (A) by *rus-1* and *rus-2* activation of the RusA resolvase and (B) by *dnaC809,820*. The strains examined are identified by genotype, and by the strain number in parentheses. C. Synthetic lethality assays demonstrating the inviability of *priB priC* cells and the restoration of viability by *dnaC809,820*. Each image is labelled as described in the legend to Fig. 2C.

Our studies revealed that eliminating PriB increases the sensitivity of Δ*ruvABC* cells to killing by UV light and reduces their ability to foster recombinants in genetic crosses. The increase in UV sensitivity approaches the synergism between *ruv* and *recG* null alleles (Fig. 2A, panels i and iii). Yields of haploid recombinants in genetic crosses are some 10-fold lower than with the *ruv* control (Tables 3A and S2). Inactivation of PriB alone has little or no effect on recombination, as reported (Sandler *et al*., 1999). The recovery of F-prime transconjugants with the *priB ruv* double mutant is reduced to an even greater extent (> 100-fold; Table 3A). Efficient zygotic induction of phage λ in the cross with Hfr GY2200 indicates that this latter defect is not due to reduced DNA transfer. Significantly, activation of the normally quiescent RusA Holliday junction resolvase *via rus-1* or *rus-2* insertions restores efficient recovery of both F-prime transconjugants and haploid recombinants (Tables 3A and S2). It also increases resistance to UV irradiation (Fig. 5A).

**Table 3:** Effect of PriB on conjugational DNA transfer and recombination

				Relative numbers of transconjugants or P1 transductants^a^				
				
				KL548	Hfr GY2200		Hfr KL226^d^	
							
	Strain number	Relevantgenotype	Relative viability^a^	(F′ Pro^+^)	(λ)^c^	(Thr^+^Leu^+^)	(Pro^+^)	P1 transductants (Leu^+^)
A	AM2077	*priB*	0.98	0.89	1.06	0.83	1.05	0.4
	N4454	*ruvABC*	0.62	0.62	0.84	0.42	0.43	0.18
	AM2078	*priB ruvABC*	0.28	0.0017	0.72	0.026	0.034	0.011
	N7946	*priB ruvABC rus-2*	0.76	1.07	1.33	0.56	0.46	0.23
B	AM2089	*priB recG*	0.77	0.85	0.97	0.69	0.69	0.49
	AM2142	*priB recB*	0.25	0.15	0.56	0.00088	0.0005	0.0042
C	AM2096	*priB ruvABC recA*	0.48	0.59	0.84	0.000024	0.000023	ND
	N7938	*priB ruvABC lexA3*	0.46	0.58	0.76	0.09	0.15	ND
	N7940	*priB ruvABC sfiA*	0.29	0.006	0.70	0.05	0.016	0.0099
	N8035	*priB ruvABC recB*	0.25	0.00008	0.93	0.00027	0.00061	ND
	AM2097	*priB ruvABC recF*	0.57	0.64	0.87	0.37	0.37	0.14
	AM2133	*priB ruvABC recJ*	0.42	0.42	0.86	0.088	0.14	0.11
	AM2134	*priB ruvABC recQ*	0.51	0.55	0.65	0.08	0.10	0.08
D	N7915	*priB ruvABC dnaC809,820*	0.61	0.85	1.16	0.25	0.56	0.19
	N7926	*priB ruvABC dnaC809,820 priC*	0.2	0.0028	0.93	0.036	0.29^e^	0.008
	N7918	*priB dnaC809,820 priC*	0.85	0.99	1.30	0.35	1.56	0.35
	N7934	*ruvABC priC*	0.52	0.68	0.58	0.27	0.47	0.14
E	N7964	*priB ruvABC rpoB[G1260D]*	1.32	0.70	0.72	0.17	0.16	0.17
	N7948	*ruvABC rpoB[G1260D]*	1.41	1.06	0.47	0.26	0.28	0.24

a. Values for cell viability are based on the recipient cultures used in conjugational crosses. Those based on cultures of the same recipients used in P1 transductions are shown in Table S2. Although the culture conditions are not the same, the two estimates are generally very close.

b. Mating was for 30 (KL548), 40 (KL226) or 60 (GY2200) min and the transconjugant class selected is indicated. The phage P1 donor was W3110. Values for wild-type control strain AB1157 are set at 1. The actual values ± SE are shown in Table S2. Mutant strains were tested in parallel with AB1157 and the values shown are mean yields relative to AB1157 in each of three or more experiments. Numbers of experiments, control mutant strains and standard errors are provided in Table S2. ND, not determined.

c. λ plaque forming units arising from zygotic induction of the λ prophage transferred by the Hfr.

d. Very similar values were obtained using N7610 as the Hfr donor, a Δ*priB*::*dhfr* derivative of Hfr KL226.

e. The Hfr transfers *priC^+^* proximal to the selected marker, hence the increased recovery of recombinant relative to the cross with Hfr GY2200, which transfers *priC^+^* distal to the selected marker such that fewer of the selected transconjugants receive this allele.

A notable feature of *ruv* mutant cells is that they foster the recovery of recombinants in genetic crosses with Hfr donors with a frequency only some two- to threefold lower than with a *ruv*^+^ control despite the lack of any other known activity capable of cleaving Holliday junctions (Table 3A) (Lloyd *et al*., 1984; Lloyd, 1991; Mandal *et al*., 1993; Mahdi *et al*., 1996). However, the viability of *ruv* cells is much reduced if the incidence of recombination is increased by exposure to UV light or other agents that damage DNA (Lloyd *et al*., 1984), or by mutations that compromise DNA macromolecular metabolism (Magner *et al*., 2007; Zhang *et al*., 2010). Viability is maintained in these circumstances if the RusA resolvase is expressed, demonstrating that the lethality observed without either resolvase is due to the accumulation of unresolved Holliday junctions (Mandal *et al*., 1993; Mahdi *et al*., 1996; Zhang *et al*., 2010). Thus, from the data presented it seems clear that PriB normally limits the incidence of recombination in conjugational crosses and during repair of UV-irradiated cells. Without PriB, recombination occurs more frequently in these situations, generating Holliday junctions. With no RuvABC available, these junctions persist, compromising viability. There is no evidence that recombination is essential in the absence of PriB. This is evident from the viability of *priB* derivatives lacking various combinations of the major activities linked with promoting recombination (Tables 3 and S2).

Eliminating PriB from *recG* cells has little effect on recombination (Table 3B). This is consistent with RuvABC acting independently of RecG (Lloyd, 1991). Importantly, a *recB* mutation reduces recombinant yields by some 200-fold or more (Table 3B), establishing that the vast majority of progeny recovered in crosses with Δ*priB* recipients are still formed via a RecBCD-dependent mechanism, as in wild-type cells.

### Homologous recombination prevents the recovery of F-prime transconjugants

RecA is essential for conjugational recombination in *E. coli*, but not for the recovery of F-prime transconjugants (Clark and Margulies, 1965). We exploited this fact to investigate whether the reduced recovery of F-prime transconjugants with *priB ruv* cells is due to abortive recombination between a newly transferred F-prime element and the recipient chromosome. We discovered that eliminating RecA restores the ability to recover F-prime transconjugants with high efficiency (Table 3C). Introducing a *lexA3* mutation, which reduces expression of RecA and prevents induction of the SOS response (Sassanfar and Roberts, 1990), also restores efficient recovery of F-prime transconjugants. However, eliminating the SOS-induced SfiA division inhibitor does not (Table 3C), from which we conclude that the failure to recover these transconjugants is not due to lethal, SOS-induced cell filamentation. Taken together, the data indicate instead that in the absence of PriB, recombination between a newly transferred F-prime and the chromosome occurs in the vast majority (≥ 99%) of transconjugants and leads to the formation of at least one Holliday junction that physically links the two DNA elements. Without RuvABC or RusA to resolve the junction, the transconjugant is inviable.

Eliminating RecF, RecO or RecR also rescues F-prime transconjugants whereas the inactivation of RecBCD enzyme does not (Tables 3C and S2). The RecFOR proteins facilitate loading of RecA on single-stranded DNA (ssDNA) bound by SSB protein. They enable RecA to displace the SSB and form a stable nucleoprotein filament that promotes homologous DNA pairing and strand exchange (Cox, 2007). Thus, the recombination provoked in the absence of PriB is most likely initiated at one or more ssDNA gaps. This would fit with the fact that during conjugation a single strand of DNA is transferred to the recipient with a 5′–3′ polarity, where it is then made duplex by lagging strand synthesis (Willetts and Wilkins, 1984; Lloyd and Buckman, 1995). The transferred donor DNA is likely therefore to contain transient ssDNA gaps that provide potential templates for the binding of PriB, SSB or both. PriB resembles SSB in several respects and is known to bind ssDNA. Our results may be explained if gaps are more common, persist for longer or are simply more recombinogenic when there is no PriB present. This would fit with our observation that inactivating RecJ or RecQ also restores a robust recovery of F-prime transconjugants (Table 3C). Without PriB to bind the transferred F-prime strand, any newly synthesized lagging strand may be targeted by a combination of the helicase activity of RecQ and the 5′–3′ ssDNA exonuclease activity of RecJ, thus delaying gap closure.

Eliminating RecFOR, RecJ or RecQ also improves slightly the recovery of haploid recombinants in Hfr crosses (Tables 3 and S2). In such crosses, it is thought that RecBCD enzyme facilitates initiation of two recombination events, one at either end of the linear Hfr DNA fragment transferred to the recipient (Smith, 1991). If true, and if single-strand gaps do persist in the transferred Hfr DNA, then it would seem that additional exchanges initiated at these gaps might be detrimental to the recovery of recombinants when the RuvABC resolvase is missing. However, we note that eliminating RecFOR, RecJ or RecQ also improves the recovery of transductants in crosses with phage P1 (Tables 3 and S2). We are unaware of any evidence to suggest that the linear fragment of duplex donor DNA in transducing particles contains single-strand interruptions that might trigger recombination.

### *dnaC809,820* promotes recovery of F-prime transconjugants, but only if PriC is present

We exploited *dnaC809,820* to examine the possibility that F-prime DNA strand transferred to a *priB* cell provokes recombination because of delayed or incomplete synthesis of the complementary (lagging) strand. The mutant DnaC protein is believed to load DnaB without the aid of PriA or PriC (Sandler, 2000). It might therefore compensate for the absence of PriB, and thus eliminate the observed synergism between *priB* and *ruv*. This proved to be the case. However, its ability to do so depends on PriC (Tables 3D and S2; Fig. 5B). The need for PriC is unexpected as *dnaC809,820* has been reported to act as a very effective suppressor of the near inviability of a *priB priC* double mutant (Sandler, 2000). A synthetic lethality assay confirmed that it does so under our experimental conditions (Fig. 5C). Deletion of *priC* alone does not reduce the recovery of either F-prime transconjugants or haploid recombinants, nor does it increase sensitivity to UV light. Unlike Δ*priB* it also does not enhance the *ruv* phenotype (Tables 3D and S2; Fig. 5B). So, while the mutant DnaC protein encoded by *dnaC809,820* is able to overcome the synergism between *priB* and *ruv* null alleles, it can do so only with the aid of PriC. We assume PriC is needed to help direct DnaB loading. With RuvABC available, *priB dnaC809,820* cells show little or no such requirement (Table 3D; Fig. 5B). From these data, we conclude that the newly transferred F-prime DNA strand provokes recombination in the absence of PriB because of a failure to initiate or complete synthesis of the complementary strand, thus increasing the likelihood of loading RecA.

### RNA polymerase mutations reduce the synergism between *priB* and *ruv*

We tested the *rpo* alleles identified as suppressors *of priB* and *priB recG* cells to see if they too might alleviate the synergism observed between *priB* and *ruv*. We found that they do. All tested alleles restore efficient recovery of F-prime transconjugants, improve the yield of haploid recombinants and reduce killing by UV light (Tables 3E and 4; Fig. S3). The improved ability to survive UV irradiation varies according to how the *rpo* allele affects the survival of *ruv* (*priB*^+^) cells, although the data reveal an imperfect correlation (Fig. S3). Nevertheless, they do indicate that the *rpo* suppressors are somehow able to reduce the incidence of recombination events that require processing by RuvABC.

**Table 4:** Effect of *rpo* suppressors of *priB* on the recovery of F-prime transconjugants in crosses with a Δ*priB* Δ*ruvABC* recipient

Strain	Suppressor	Relative yield of F-prime transconjugants^a^
AM2078	None	0.0017
N8174	*rpoA[S49T, S309P]*	0.28 ± 0.07
N8175	*rpoA[E273D]*	0.30 ± 0.02
N8185	*rpoA[K298N]*	0.17 ± 0.03
N8187	*rpoA[L253R]*	0.14 ± 0.06
N8004	rpoB^*^35[H1244Q]	0.4 ± 0.03
N8179	*rpoB[*Δ*D446-L448]*	0.34 ± 0.04
N8180	*rpoB[S1332L]*	0.37 ± 0.05
N8181	*rpoB[R452L]*	0.21 ± 0.09
N8178	*rpoC[*Δ*K215-R220]*	0.28 ± 0.03

a. Values are relative to the yield with the wild-type (*pri^+^ ruv^+^*) control strain, AB1157, and are the means (± SE) of from three to five independent experiments.

### The *rpoA*, *rpoB* and *rpoC* mutations improve the viability of *priB polA* cells

**Fig. 6:**
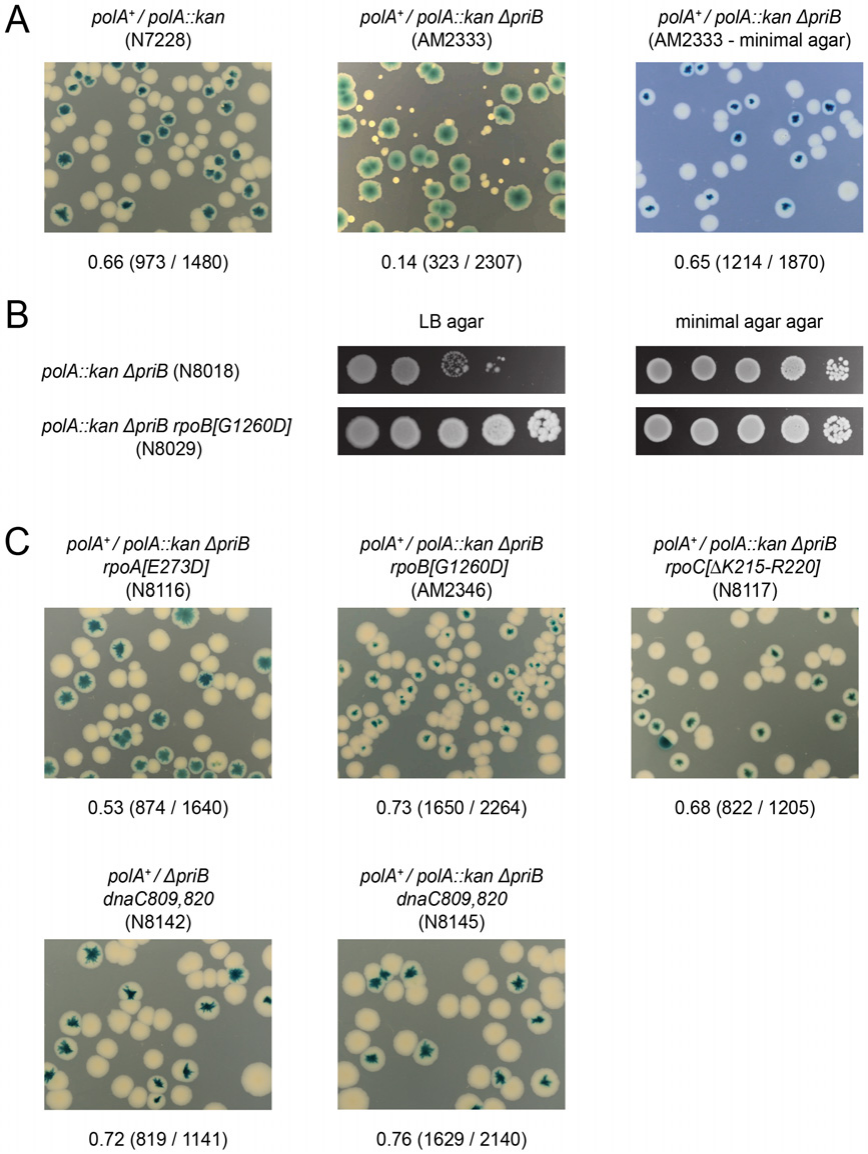
RNA polymerase and DnaC mutations improve the viability of *polA priB* cells. A. Synthetic lethality of *polA priB* cells. Each assay is labelled as described in the legend to Fig. 2C. B. Relative plating efficiency of *polA priB* cells on LB and minimal salts agar. The strains indicated were derived using 56/2 minimal salts agar media. Single colonies were grown in liquid 56/2 salts to an *A*_650_ of 0.4, serially diluted in 10-fold steps from 10^−1^ to 10^−5^ and 10 μl of samples of each dilution were spotted on LB or minimal salts agar as indicated. Plates were photographed after 48 h incubation. C. Synthetic lethality assays demonstrating robust growth on LB agar of *polA priB* cells carrying the indicated *rpoA* alleles or *dnaC809,820*. Each image is labelled as described in the legend to Fig. 2C.

Our analysis of *priB* cells revealed that PriB is required to help maintain viability in the absence of DNA polymerase I, at least under conditions supporting rapid growth. Without it, these cells plate with high efficiency on minimal salts agar, but are able to establish many fewer and rather sickly colonies on LB agar (Fig. 6A and B). This finding is not that surprising given that these cells have been shown to require the PriA-dependent pathway of replication restart to maintain viability (Lee and Kornberg, 1991). The *rpo* suppressors of *priB* we have identified allow robust growth of *priB polA* cells on LB agar (Fig. 6B and C. This observation provides further support for the conclusion that the suppression of *priB* by the *rpo* alleles described is not limited to the elimination of sensitivity to mitomycin C, reinforcing the conclusion that the latter effect is not some consequence of changes in gene expression that reduce the uptake of mitomycin C or which increase its efflux.

## Discussion

We identified a novel suppressor of *recG* as a nonsense mutation in the *rpsF* gene encoding ribosomal subunit S6 (*rpsF292*; Fig. 1A). Because of its location upstream of *priB*, we thought it might act by exerting a polar effect, reducing synthesis of PriB and thus compromising DnaB loading. In other words, we suspected it might have an effect similar to previously identified *priA* suppressors that reduce the helicase activity of PriA (Al-Deib *et al*., 1996; Gregg *et al*., 2002; Jaktaji and Lloyd, 2003; Zhang *et al*., 2010). We dissected the contributions of *rpsF* and *priB* and demonstrated that a reduction in PriB synthesis might indeed be a substantial factor. However, the analysis revealed that the mutation of RpsF itself also makes a contribution (Fig. 1C). Indeed, we showed that an in-frame deletion of *rpsF* has suppressor activity (Fig. 3A and B).

The conclusion that reduced expression of *priB* is not by itself sufficient to explain the effect of *rpsF292* is re-enforced by finding that a *priB* deletion is a weak suppressor of the mitomycin C sensitivity conferred by *recG*. However, this is not surprising as the deletion itself confers some sensitivity, and has other debilitating effects (see below). Intriguingly, the *priB* deletion becomes much more effective in the presence of an additional mutation in one of three major subunits of RNA polymerase. The mutations identified alleviate every aspect of the deletion *priB* phenotype we have tested, including the sensitivity to mitomycin C (Fig. 4A), the synergism with *ruv* (Table 3; Fig. 2) and the inviability with *polA* (Fig. 6). Although conditional, the fact that the absence of PriB can be a very effective suppressor is consistent with the view that much of the *recG* pathology is due to over-replication of the chromosome following PriA-mediated replisome assembly (Rudolph *et al*., 2009b; 2010a,2010b).

Analysis of the synergism with *ruv* revealed a strong tendency in cells lacking PriB for recombination to be provoked. However, this recombination is not essential, as is clear from the viability of deletion *priB* cells lacking RecA, RecBCD or RuvABC (Table 3). Analysis of the factors that eliminate the synergism with *ruv* indicated that recombination is provoked because one or more regions of ssDNA become exposed to RecA (Table 3, Fig. 5). We assume this occurs when the PriA–PriB–DnaT system is recruited to facilitate replisome assembly. PriB normally limits exposure of ssDNA by binding to the ssDNA exposed by PriA before transferring it via DnaT to the DnaC:DnaB complex (Lopper *et al*., 2007).

Our finding that mutations in RNA polymerase suppress the deletion *priB* phenotype would be consistent with the idea that PriB plays an important part in resolving conflicts between DNA replication and transcription. But if true, how could a deficiency in this activity be reconciled with the ability of deletion *priB* to suppress *recG*. Transcription complexes are substantial barriers to replication fork progression (Mirkin and Mirkin, 2007; Rudolph *et al*., 2007a; Merrikh *et al*., 2011), and may be particularly troublesome if they stall or backtrack (Trautinger *et al*., 2005; Dutta *et al*., 2011). Several recent studies indicate that recruitment of a second helicase motor helps drive forks through these barriers and that viability is compromised if the primary candidates are not available, as for example in *rep uvrD* strains (Guy *et al*., 2009; Baharoglu *et al*., 2010; Boubakri *et al*., 2010; Atkinson *et al*., 2011). Viability is improved by *rpoB* and *rpoC* mutations that destabilize transcribing RNA polymerases. Therefore, our finding that many features of the *priB* null phenotype are suppressed by some of the very same *rpo* mutations is highly significant, and especially so given *dnaC809,820* is also a suppressor. It suggests that replication forks not only stall when they run into RNA polymerase, but also frequently require the re-loading of DnaB before replication can resume.

A need to re-load DnaB explains how a combination of *priB* and *rpo* mutations strongly suppresses *recG*. Assuming the *recG* phenotype is a consequence of PriA-dependent chromosome over-replication, as the results presented would suggest, it would be reasonable to suppose that this replication increases conflicts with transcription, especially if it were to initiate in the terminus area and proceed towards *oriC*, as suggested (Rudolph *et al*., 2010b). Eliminating PriB would prevent this over-replication by disrupting the replisome assembly needed for its initiation, while destabilizing RNA polymerase would itself reduce the need for PriB to rescue those forks assembled initially at *oriC* that subsequently ran into trouble. With PriB present in *rpo recG* cells, the over-replication triggered in the absence of RecG would negate any advantage gained from destabilizing RNA polymerase, thereby explaining the failure of the *rpo* mutation itself to suppress *recG*.

The RNA polymerase mutations implicated in reducing conflicts between replication and transcription most probably do so by reducing the stability of transcription complexes, thereby reducing the barrier to replication fork progression. In the case of *rpoC[*Δ*K215-R220]* and rpoB*35, destabilization has been demonstrated experimentally, and most likely reflects the disruption of important stabilizing interactions in the DNA channel (Bartlett *et al*., 1998; Trautinger *et al*., 2005). However, the *rpoA* alleles identified seem unlikely to compromise the intrinsic stability of RNA polymerase. With the exception of the S49T substitution encoded by *rpoA[S49T,S309P]*, all affect the mobile C-terminal domain of the RpoA (alpha) subunit that interacts with NusA and with the emerging mRNA (Mah *et al*., 2000). The E273D, P293L and K298N substitutions may directly affect binding to NusA. The L253R and S309P substitutions are distant to the NusA binding interface, but might affect the total mobility of the domain and thus indirectly affect the interaction. NusA binding to RNA polymerase affects the β-flap domain of the RNA exit channel, exerting an allosteric effect on the trigger loop/bridge helix interaction required for translocation of the elongation complex, thus reducing elongation and increasing pausing (Bar-Nahum *et al*., 2005; Nudler, 2009). If the *rpoA* alleles reduce NusA binding, they might therefore destabilize transcription complexes indirectly by reducing pausing and uncoupling transcription from translation, enabling Rho to unwind the untranslated RNA (Epshtein *et al*., 2010; Dutta *et al*., 2011; Washburn and Gottesman, 2011). The idea that Rho might be a critical factor in reducing conflicts between replication and transcription is consistent with the reported synthetic lethality of *recG rho* double mutant cells (Harinarayanan and Gowrishankar, 2003), and with the identification here of ribosomal subunit S6 mutations as suppressors of *recG*. It may also be significant that the conditional *rho-15* allele confers methionine auxotrophy (Guterman and Howitt, 1979), a property shared with *rpoA[P293L]*, the most frequent suppressor in our screens for *priB recG* derivatives resistant to mitomycin C. If our interpretation is correct, it would follow that by coupling transcription with translation, and thus reducing Rho-mediated termination, the presence of NusA actually increases conflicts with replication. We assume that premature termination of transcription is a more immediate threat to growth and viability than is presented by blocking replication fork progression.

To conclude, we have identified novel suppressors of the *recG* mutant phenotype that combine a deficiency in the PriB component of the PriA–PriB–DnaT system of replisome assembly with modifications either to the ribosome or to RNA polymerase. By dissecting the properties of these suppressors and probing their modes of action, we have confirmed that the pathology resulting from loss of RecG is largely a consequence of unscheduled chromosome replication mediated by the PriA–PriB–DnaT system of replisome assembly. We have also presented evidence that this replication most likely increases conflicts with transcription and that PriB is needed to help resolve such conflicts. Eliminating PriB suppresses *recG*, presumably by reducing unscheduled replication, but only in the presence of an additional mutation to RNA polymerase that is itself likely to reduce conflicts between replication and transcription. The RNA polymerase mutations identified include *rpoA* alleles likely to disrupt interactions with NusA, leading us to suspect that factors controlling the coupling of transcription and translation may play a significant role in balancing the different pressures on replication and transcription.

## Experimental procedures

### Bacterial strains

The strains used are listed in Table S1. Chromosomal genes were inactivated using Tn*10* or *kan* insertions conferring resistance to tetracycline (Tc^r^) and kanamycin (Km^r^), respectively, or with deletions tagged with insertions conferring resistance to chloramphenicol (*cat*; Cm^r^), kanamycin (*kan*; Km^r^), trimethoprim (*dhfr*; Tm^r^) or apramycin (*apra*; Apra^r^). The Δ*priB202* allele is an in-frame deletion of the *priB*-coding sequence (Sandler *et al*., 1999). It was introduced by co-transduction with *zjf920*::Tn*10*. A new in-frame deletion (Δ*priB*::*dhfr*) was made using the one-step gene inactivation method of Datsenko and Wanner (2000). The entire *priB* sequence from start to stop codon was replaced with a *dhfr* sequence. The same method was used to make an in-frame deletion of *rpsF* (Δ*rpsF*::*cat*) and internal deletions of *dam* (Δ*dam*::*dhfr*) and *recR* (Δ*recR*::*kan*). The *dam* deletion leaves 42 bp of coding sequence at the 5′ end and 48 bp at the 3′ end while the *recR* deletion leaves 96 bp 5′ and 51 bp 3′. The *yheB*::*kan* and *yheR*::*kan* insertion alleles linked to *rpoA*, and the *mutL*::*kan* allele linked to *rpsF*, were identified using a library of random *kan* insertions in strain MG1655 generated using the EZ-Tn*5* <kan-2> Tnp Transposome system (Epicentre Technologies). Neither of the *yhe* insertions has any obvious effect on growth or sensitivity to genotoxic agents (R.G. Lloyd, unpubl. work).

### Plasmids

pRC7 is a low-copy-number, mini-F derivative of the *lac^+^* construct pFZY1 (Bernhardt and de Boer, 2004). pJJ100 and pAM475 are derivatives of pRC7 carrying *recG^+^* and *polA^+^* respectively (Zhang *et al*., 2010). A *priC^+^* derivative was made by PCR amplification of the coding region for *priC* from strain MG1655, plus some 100 bp of upstream promoter sequences, using 5′ and 3′ primers that incorporated flanking ApaI restriction sites. The amplified DNA was cut with ApaI and the *priC^+^* fragment inserted into the ApaI site within the *lacI^q^* gene of pRC7, generating pAM421. This plasmid maintains robust growth of a Δ*priC* Δ*priB* strain, demonstrating that it expresses *priC^+^*. pT7 cloning vectors have been described (Tabor and Richardson, 1985). pAM494 is a derivative of pT7-7 carrying the adjacent *rpsR^+^* and *rplI^+^* genes inserted between the vector NdeI and HindIII sites. pAM496 and pAM499 are equivalent constructs carrying *priB^+^* and *rpsF^+^* respectively. pGB061 is an *rpsF^+^* derivative of the expression vector pTRc99a (Amann *et al*., 1988). Expression of *rpsF* in strains harbouring pGB061 was induced by growth in LB media containing 0.15 mM IPTG. Media were supplemented with ampicillin for plasmid maintenance, except as specified in synthetic lethality assays with strains carrying pRC7 and its derivatives.

### Media and general methods

LB broth and 56/2 minimal salts media, and methods for monitoring cell growth and for strain construction by P1*vir*-mediated transduction have been cited (Al-Deib *et al*., 1996; McGlynn and Lloyd, 2000; Trautinger *et al*., 2005). Resistance to rifampicin was measured by streaking culture samples on LB agar plates supplemented with rifampicin at a final concentration of 5, 10, 15, 20 and 50 μg ml^−1^ and scoring growth after overnight incubation.

### Isolating mitomycin C-resistant suppressors of Δ*recG* and Δ*recG* Δ*priB* strains

*E. coli* strains lacking RecG, or both RecG and PriB, are sensitive to mitomycin C. Several independent cultures of these strains were set up from single colonies and grown to mid-exponential phase in LB broth before plating 50–100 μl of samples on LB agar plates supplemented with mitomycin C at a final concentration of 0.5 μg ml^−1^. Resistant mutants establishing robust colonies appear within 24–36 h at 37°C. They arise at a frequency of approximately 0.1–1 per 10^6^ colony-forming units (cfu) plated.

### Measuring sensitivity to DNA damage

Sensitivity to UV light was measured using exponential phase cells grown to an *A*_650_ of 0.4 (1–2 × 10^8^ cells ml^−1^ for strain MG1655). Samples of appropriate dilutions were irradiated on the surface of LB agar plates and survivors scored after 18–24 h incubation. Survival data are means from at least two, usually 3–6, independent experiments. Errors (SE) range between 5% and 15% of the mean. Sensitivity to mitomycin C (MC) was determined by growing cultures to an *A*_650_ of 0.4 and spotting 10 μl of serial 10-fold dilutions from 10^−1^ to 10^−5^ (from left to right in the images shown) on LB agar with or without mitomycin C at a final concentration of 0.5 μg ml^−1^ and incubating at 37°C, with or without prior exposure to UV light, as indicated. Plates were photographed after 24 h incubation, unless stated otherwise. Media contained ampicillin at a final concentration of 50 μg ml^−1^ in the case of strains harbouring Ap^r^ plasmids.

### Multiplication of cells surviving UV irradiation

Cultures of each strain were grown in LB both to an *A*_650_ of 0.2, the cells pelleted, UV-irradiated or mock-irradiated on the surface of LB agar and resuspended in the original, but filter-sterilized supernatant and diluted 10 000-fold in conditioned medium prepared by growing the wild-type strain in fresh LB broth to an *A*_650_ of 0.2 with subsequent filter sterilization. The diluted cells were incubated with vigorous aeration at 37°C and samples removed at intervals were mixed with 2.5 ml of molten 0.6% top agar and plated on LB agar. Colonies were scored after 18–24 h at 37°C.

### Genetic crosses and measures of recombination

F-prime and Hfr donors were mated with F^−^ recipient strains in high-salt LB broth at 37°C as described (Lloyd *et al*., 1987; 1988). Measurements of cell viability relate to the number of cfu in the recipient culture at an *A*_650_ of 0.4, as determined with plating on non-selective 56/2 agar. All recipients were derivatives of the multi-auxotrophic, streptomycin-resistant strain, AB1157 (Table S1). Transconjugants were selected using 56/2 or LB agar, as appropriate, supplemented with 100 μg ml^−1^ streptomycin to counterselect donor cells. Transductions were conducted using phage P1 *vir*, following the recipes and protocols described (Miller, 1972).

### Synthetic lethality assays

The rationale for synthetic lethality assays has been described (Bernhardt and de Boer, 2004; Mahdi *et al*., 2006). Essentially, a wild-type gene of interest is cloned in pRC7, a *lac^+^*, Ap^r^ mini-F plasmid that is rapidly lost, and used to cover a null mutation in the chromosome, in a Δ*lac* background. A mutation in another gene of interest is then introduced into the chromosome. If the double mutant is viable, the plasmid-free cells segregated during culture will form white (Lac^−^) colonies or sectors of colonies on agar plates supplemented with X-gal and IPTG. If synthetically lethal, they will fail to grow and only solid blue (Lac^+^) colonies formed by cells retaining the plasmid will be observed. The segregation of white colonies that are significantly smaller than blue colonies is generally an indicator of reduced viability without the covering plasmid. Cultures of the constructs tested were grown in LB broth without ampicillin selection to an *A*_650_ of 0.4 before assaying for growth of plasmid-free cells on indicator plates. Plates were photographed after incubation for 48 h (LB agar) or 72 h (glucose minimal salts agar). Photographs were cropped to show a 3 cm × 2 cm section of the plate agar. Unless stated otherwise, images are from LB indicator plates.

## References

[b1] Al-Deib AA, Mahdi AA, Lloyd RG (1996). Modulation of recombination and DNA repair by the RecG and PriA helicases of *Escherichia coli* K-12. J Bacteriol.

[b2] Amann E, Ochs B, Abel KJ (1988). Tightly regulated *tac* promoter vectors useful for the expression of unfused and fused proteins in *Escherichia coli*. Gene.

[b3] Asai T, Kogoma T (1994a). D-loops and R-loops: alternative mechanisms for the initiation of chromosome replication in *Escherichia coli*. J Bacteriol.

[b4] Asai T, Kogoma T (1994b). Roles of *ruvA, ruvC* and *recG* gene functions in normal and DNA damage-inducible replication of the *Escherichia coli* chromosome. Genetics.

[b5] Atkinson J, Gupta MK, Rudolph CJ, Bell H, Lloyd RG, McGlynn P (2011). Localization of an accessory helicase at the replisome is critical in sustaining efficient genome duplication. Nucleic Acids Res.

[b6] Baharoglu Z, Lestini R, Duigou S, Michel B (2010). RNA polymerase mutations that facilitate replication progression in the *rep uvrD recF* mutant lacking two accessory replicative helicases. Mol Microbiol.

[b7] Bar-Nahum G, Epshtein V, Ruckenstein AE, Rafikov R, Mustaev A, Nudler E (2005). A ratchet mechanism of transcription elongation and its control. Cell.

[b8] Bartlett MS, Gaal T, Ross W, Gourse RL (1998). RNA polymerase mutants that destabilize RNA polymerase-promoter complexes alter NTP-sensing by *rrn* P1 promoters. J Mol Biol.

[b9] Bernhardt TG, de Boer PA (2004). Screening for synthetic lethal mutants in *Escherichia coli* and identification of EnvC (YibP) as a periplasmic septal ring factor with murein hydrolase activity. Mol Microbiol.

[b10] Boubakri H, de Septenville AL, Viguera E, Michel B (2010). The helicases DinG, Rep and UvrD cooperate to promote replication across transcription units *in vivo*. EMBO J.

[b11] Bubunenko M, Baker T, Court DL (2007). Essentiality of ribosomal and transcription antitermination proteins analyzed by systematic gene replacement in *Escherichia coli*. J Bacteriol.

[b12] Cadman CJ, Lopper M, Moon PB, Keck JL, McGlynn P (2005). PriB stimulates PriA helicase via an interaction with single-stranded DNA. J Biol Chem.

[b13] Cashel M, Gentry DR, Hernandez VJ, Vinella D, Neidhardt FC, Curtiss R, Ingraham JL, Lin ECC, Low KB, Magasanik B (1996). The Stringent Response. In: *Escherichia coli* and *Salmonella* Cellular and Molecular Biology.

[b14] Clark AJ, Margulies AD (1965). Isolation and characterization of recombination deficient mutants of *Escherichia coli* K12. Proc Natl Acad Sci USA.

[b15] Cox MM (2007). Regulation of bacterial RecA protein function. Crit Rev Biochem Mol Biol.

[b16] Datsenko KA, Wanner BL (2000). One-step inactivation of chromosomal genes in *Escherichia coli* K-12 using PCR products. Proc Natl Acad Sci USA.

[b17] Dutta D, Shatalin K, Epshtein V, Gottesman ME, Nudler E (2011). Linking RNA polymerase backtracking to genome instability in *E. coli*. Cell.

[b18] Epshtein V, Dutta D, Wade J, Nudler E (2010). An allosteric mechanism of Rho-dependent transcription termination. Nature.

[b19] Fukuoh A, Iwasaki H, Ishioka K, Shinagawa H (1997). ATP-dependent resolution of R-loops at the ColE1 replication origin by *Escherichia coli* RecG protein, a Holliday junction-specific helicase. EMBO J.

[b20] Gabbai CB, Marians KJ (2010). Recruitment to stalled replication forks of the PriA DNA helicase and replisome-loading activities is essential for survival. DNA Repair (Amst).

[b21] Gregg AV, McGlynn P, Jaktaji RP, Lloyd RG (2002). Direct rescue of stalled DNA replication forks via the combined action of PriA and RecG helicase activities. Mol Cell.

[b22] Guterman SK, Howitt CL (1979). Rho and ribosome mutation interaction: lethality of *rho-15* in *rpsL* or *rpsE* strains, and *rho-15* methionine auxotrophy in *rps+* strains of *Escherichia coli*. Genetics.

[b23] Guy CP, Atkinson J, Gupta MK, Mahdi AA, Gwynn EJ, Rudolph CJ (2009). Rep provides a second motor at the replisome to promote duplication of protein-bound DNA. Mol Cell.

[b24] Harinarayanan R, Gowrishankar J (2003). Host factor titration by chromosomal R-loops as a mechanism for runaway plasmid replication in transcription termination-defective mutants of *Escherichia coli*. J Mol Biol.

[b25] Heller RC, Marians KJ (2005). The disposition of nascent strands at stalled replication forks dictates the pathway of replisome loading during restart. Mol Cell.

[b26] Hong X, Cadell GW, Kogoma T (1995). *Escherichia coli* RecG and RecA proteins in R-loop formation. EMBO J.

[b27] Horiuchi T, Maki H, Sekiguchi M (1984). RNase H-defective mutants of *Escherichia coli*: a possible discriminatory role of RNase H in initiation of DNA replication. Mol Gen Genet.

[b28] Jaktaji RP, Lloyd RG (2003). PriA supports two distinct pathways for replication restart in UV-irradiated *Escherichia coli* cells. Mol Microbiol.

[b29] Kang WK, Icho T, Isono S, Kitakawa M, Isono K (1989). Characterization of the gene *rimK* responsible for the addition of glutamic acid residues to the C-terminus of ribosomal protein S6 in *Escherichia coli* K12. Mol Gen Genet.

[b30] Kim S, Dallmann HG, McHenry CS, Marians KJ (1996a). Coupling of a replicative polymerase and helicase: a tau–DnaB interaction mediates rapid replication fork movement. Cell.

[b31] Kim S, Dallmann HG, McHenry CS, Marians KJ (1996b). tau couples the leading- and lagging-strand polymerases at the *Escherichia coli* DNA replication fork. J Biol Chem.

[b32] Kogoma T, Cadwell GW, Barnard KG, Asai T (1996). The DNA replication priming protein, PriA, is required for homologous recombination and double-strand break repair. J Bacteriol.

[b33] LeBowitz JH, McMacken R (1986). The *Escherichia coli dnaB* replication protein is a DNA helicase. J Biol Chem.

[b34] Lee EH, Kornberg A (1991). Replication deficiencies in *priA* mutants of *Escherichia coli* lacking the primosomal replication n′ protein. Proc Natl Acad Sci USA.

[b35] Liu J, Xu L, Sandler SJ, Marians KJ (1999). Replication fork assembly at recombination intermediates is required for bacterial growth. Proc Natl Acad Sci USA.

[b36] Lloyd RG (1991). Conjugational recombination in resolvase-deficient *ruvC* mutants of *Escherichia coli* K-12 depends on *recG*. J Bacteriol.

[b38] Lloyd RG, Buckman C (1995). Conjugational recombination in *Escherichia coli*: genetic analysis of recombinant formation in Hfr x F^−^ crosses. Genetics.

[b37] Lloyd RG, Benson FE, Shurvinton CE (1984). Effect of *ruv* mutations on recombination and DNA repair in *Escherichia coli* K12. Mol Gen Genet.

[b39] Lloyd RG, Evans NP, Buckman C (1987). Formation of recombinant *lacZ*^+^ DNA in conjugational crosses with a *recB* mutant of *Escherichia coli* K12 depends on *recF, recJ*, and *recO*. Mol Gen Genet.

[b40] Lloyd RG, Porton MC, Buckman C (1988). Effect of *recF, recJ, recN, recO* and *ruv* mutations on ultraviolet survival and genetic recombination in a *recD* strain of *Escherichia coli* K-12. Mol Gen Genet.

[b41] Lopper M, Boonsombat R, Sandler SJ, Keck JL (2007). A hand-off mechanism for primosome assembly in replication restart. Mol Cell.

[b48] McCool JD, Ford CC, Sandler SJ (2004). A *dnaT* mutant with phenotypes similar to those of a *priA2:kan* mutant in *Escherichia coli* K-12. Genetics.

[b50] McGlynn P, Lloyd RG (2000). Modulation of RNA polymerase by (p)ppGpp reveals a RecG-dependent mechanism for replication fork progression. Cell.

[b51] McGlynn P, Lloyd RG (2001). Rescue of stalled replication forks by RecG: simultaneous translocation on the leading and lagging strand templates supports an active DNA unwinding model of fork reversal and Holliday junction formation. Proc Natl Acad Sci USA.

[b49] McGlynn P, Al-Deib AA, Liu J, Marians KJ, Lloyd RG (1997). The DNA replication protein PriA and the recombination protein RecG bind D-loops. J Mol Biol.

[b42] Magner DB, Blankschien MD, Lee JA, Pennington JM, Lupski JR, Rosenberg SM (2007). RecQ promotes toxic recombination in cells lacking recombination intermediate-removal proteins. Mol Cell.

[b43] Mah TF, Kuznedelov K, Mushegian A, Severinov K, Greenblatt J (2000). The alpha subunit of *E. coli* RNA polymerase activates RNA binding by NusA. Genes Dev.

[b45] Mahdi AA, Sharples GJ, Mandal TN, Lloyd RG (1996). Holliday junction resolvases encoded by homologous *rusA* genes in *Escherichia coli* K-12 and phage 82. J Mol Biol.

[b44] Mahdi AA, Buckman C, Harris L, Lloyd RG (2006). Rep and PriA helicase activities prevent RecA from provoking unnecessary recombination during replication fork repair. Genes Dev.

[b46] Mandal TN, Mahdi AA, Sharples GJ, Lloyd RG (1993). Resolution of Holliday intermediates in recombination and DNA repair: indirect suppression of *ruvA, ruvB* and *ruvC* mutations. J Bacteriol.

[b47] Masai H, Asai T, Kubota Y, Arai K, Kogoma T (1994). *Escherichia coli* PriA protein is essential for inducible and constitutive stable DNA replication. EMBO J.

[b52] Merrikh H, Machon C, Grainger WH, Grossman AD, Soultanas P (2011). Co-directional replication–transcription conflicts lead to replication restart. Nature.

[b53] Messer W (2002). The bacterial replication initiator DnaA. DnaA and *oriC*, the bacterial mode to initiate DNA replication. FEMS Microbiol Rev.

[b80] von Meyenburg K, Boye E, Skarstad K, Koppes L, Kogoma T (1987). Mode of initiation of constitutive stable DNA replication in RNase H-defective mutants of *Escherichia coli* K-12. J Bacteriol.

[b54] Miller JH (1972). Experiments in Molecular Genetics.

[b55] Mirkin EV, Mirkin SM (2007). Replication fork stalling at natural impediments. Microbiol Mol Biol Rev.

[b56] Nudler E (2009). RNA polymerase active center: the molecular engine of transcription. Annu Rev Biochem.

[b58] Nurse P, Zavitz KH, Marians KJ (1991). Inactivation of the *Escherichia coli* PriA DNA replication protein induces the SOS response. J Bacteriol.

[b57] Nurse P, Liu J, Marians KJ (1999). Two modes of PriA binding to DNA. J Biol Chem.

[b59] Ogawa T, Pickett GG, Kogoma T, Kornberg A (1984). RNase H confers specificity in the dnaA-dependent initiation of replication at the unique origin of the *Escherichia coli* chromosome *in vivo* and *in vitro*. Proc Natl Acad Sci USA.

[b60] Reeh S, Pedersen S (1979). Post-translational modification of *Escherichia coli* ribosomal protein S6. Mol Gen Genet.

[b61] Rudolph CJ, Dhillon P, Moore T, Lloyd RG (2007a). Avoiding and resolving conflicts between DNA replication and transcription. DNA Repair (Amst).

[b65] Rudolph CJ, Upton AL, Lloyd RG (2007b). Replication fork stalling and cell cycle arrest in UV-irradiated *Escherichia coli*. Genes Dev.

[b64] Rudolph CJ, Upton AL, Harris L, Lloyd RG (2009a). Pathological replication in cells lacking RecG DNA translocase. Mol Microbiol.

[b66] Rudolph CJ, Upton AL, Lloyd RG (2009b). Replication fork collisions cause pathological chromosomal amplification in cells lacking RecG DNA translocase. Mol Microbiol.

[b62] Rudolph CJ, Mahdi AA, Upton AL, Lloyd RG (2010a). RecG protein and single-strand DNA exonucleases avoid cell lethality associated with PriA helicase activity in *Escherichia coli*. Genetics.

[b63] Rudolph CJ, Upton AL, Briggs GS, Lloyd RG (2010b). Is RecG a general guardian of the bacterial genome?. DNA Repair (Amst).

[b67] Sandler SJ (2000). Multiple genetic pathways for restarting DNA replication forks in *Escherichia coli* K-12. Genetics.

[b68] Sandler SJ, Marians KJ (2000). Role of PriA in replication fork reactivation in *Escherichia coli*. J Bacteriol.

[b70] Sandler SJ, Samra HS, Clark AJ (1996). Differential suppression of *priA2:kan* phenotypes in *Escherichia coli* K-12 by mutations in *priA, lexA*, and *dnaC*. Genetics.

[b69] Sandler SJ, Marians KJ, Zavitz KH, Coutu J, Parent MA, Clark AJ (1999). *dnaC* mutations suppress defects in DNA replication- and recombination-associated functions in *priB* and *priC* double mutants in *Escherichia coli* K-12. Mol Microbiol.

[b71] Sassanfar M, Roberts JW (1990). Nature of the SOS-inducing signal in *Escherichia coli*. The involvement of DNA replication. J Mol Biol.

[b72] Smith GR (1991). Conjugational recombination in *E. coli*: Myths and mechanisms. Cell.

[b73] Tabor S, Richardson CC (1985). A bacteriophage T7 RNA polymerase/promoter system for controlled exclusive expression of specific genes. Proc Natl Acad Sci USA.

[b74] Tanaka T, Masai H (2006). Stabilization of a stalled replication fork by concerted actions of two helicases. J Biol Chem.

[b75] Thomas MS, Glass RE (1991). *Escherichia coli rpoA* mutation which impairs transcription of positively regulated systems. Mol Microbiol.

[b76] Tougu K, Peng H, Marians KJ (1994). Identification of a domain of *Escherichia coli* primase required for functional interaction with the DnaB helicase at the replication fork. J Biol Chem.

[b78] Trautinger BW, Lloyd RG (2002). Modulation of DNA repair by mutations flanking the DNA channel through RNA polymerase. EMBO J.

[b77] Trautinger BW, Jaktaji RP, Rusakova E, Lloyd RG (2005). RNA polymerase modulators and DNA repair activities resolve conflicts between DNA replication and transcription. Mol Cell.

[b79] Vincent SD, Mahdi AA, Lloyd RG (1996). The RecG branch migration protein of *Escherichia coli* dissociates R-loops. J Mol Biol.

[b81] Washburn RS, Gottesman ME (2011). Transcription termination maintains chromosome integrity. Proc Natl Acad Sci USA.

[b82] Willetts N, Wilkins B (1984). Processing of plasmid DNA during bacterial conjugation. Microbiol Rev.

[b83] Zhang J, Mahdi AA, Briggs GS, Lloyd RG (2010). Promoting and avoiding recombination: contrasting activities of the *Escherichia coli* RuvABC Holliday junction resolvase and RecG DNA translocase. Genetics.

