# Supplementary material for: Modulation of DNA damage tolerance in Escherichia coli recG and ruv strains by mutations affecting PriB, the ribosome and RNA polymerase
Source: Mol Microbiol. 2012 Sep 7;86(3):675–91. doi: 10.1111/mmi.12010 (PMC3533792; doi:10.1111/mmi.12010)
Supplement: Supplementary file 1 [file mmi0086-0675-SD1.pdf]

SUPPORTING INFORMATION

Modulation of DNA damage tolerance in *Escherichia coli recG* strains by mutations affecting the ribosome, RNA polymerase and replisome assembly

Akeel A. Mahdi, Geoffrey S. Briggs and Robert G. Lloyd*

Institute of Genetics, University of Nottingham, Queen's Medical Centre,

Nottingham, NG7 2UH, United Kingdom

Supporting Material

Table S1. *Escherichia coli* K-12 strains

Strain	Relevant Genotype[a]	Source or reference
**Hfr and F-prime strains, and P1 donors**		
AM2054	*ΔpriC303::kan ΔpriB202 dnaC809,820 hisG4 argE3 rpsL sulA*::Mu*d*Ap*lac*MuB::Tn*9 Δ(lac-pro)*$_{x111}$ *zjf-920*::Tn*10*	JJC1405 x P1.CGSC7484 to Tc^r
CGSC7484	*zjf-920*::Tn*10*	CGSC[b]
CGSC7488	*zji-202*::Tn*10*	CGSC
GY2200	Hfr (H, PO1) (λ*ind*)$^+$ *thi-1 relA1*	R. Devoret
JC12334	*tnaA*::Tn*10 recF143*	A. J. Clark
JJC1405	*ΔpriC303::kan ΔpriB202 dnaC809,820 hisG4 argE3 rpsL sulA*::Mu*d*Ap*lac*MuB::Tn*9 Δ(lac-pro)*$_{x111}$	Steve Sandler strain via Bénédicte Michel
KL226	Hfr (Cavalli, PO2A) *relA1 tonA22*	K. B. Low
KL227	Hfr (PO3 of P4X) *metB1*	K.B. Low
KL548	F' (F128) *lacI3 lacZ118 proAB*$^+$	K. B. Low
N2231	*malE*::Tn*10*	(Picksley *et al.*, 1984)
N3005	*purE85*::Tn*10*	(Lloyd and Buckman, 1985)
N3072	*recA269*::Tn*10*	(Lloyd *et al.*, 1987)
N4452	*ΔrecG265::cat*	(Jaktaji and Lloyd, 2003)
N6424	*ΔpriC303::kan ΔpriB202 dnaC809,820 hisG4 argE3 rpsL sulA*::Mu*d*Ap*lac*MuB::Tn*9 Δ(lac-pro)*$_{x111}$ *zji-202*::Tn*10*	JJC1405 x P1.CGSC7488 to Tc^r
N7610	Hfr (Cavalli, PO2A) *relA1 tonA22 ΔpriB::dhfr*	KL226 x P1.AM2013 to Tm^r
TRM308	*recB268*::Tn*10*	(Mahdi *et al.*, 2006)
W3110	IN(*rrnD-rrnE)1*	(Bachmann, 1996)
**AB1157 and derivatives**		
AB1157	F$^-$ λ$^-$ rac$^-$ *thi-1 hisG4 Δ(gpt-proA)62 argE3 thr-1 leuB6 kdgK51 rfbD1 araC14 lacY1 galK2 xylA5 mtl-1 tsx-33 supE44(glnV44) rpsL31*(Str^r)	(Bachmann, 1996)
AM2077	*ΔpriB::dhfr*	AB1157 x P1.AM2013 to Tm^r
AM2078	*ΔruvABC::cat ΔpriB::dhfr*	N4454 x P1.AM2013 to Tm^r
AM2089	*ΔpriB::dhfr ΔrecG::apra*	AM2077 x P1.N6052 to Apra^r
AM2094	*ΔruvABC::cat recA269*::Tn*10*	N4454 x P1.N3072 to Tc^r
AM2095	*ΔruvABC::cat Δ tnaA*::Tn*10 recF143*	N4454 x P1.JC12334 to Tc^r
AM2096	*ΔruvABC::cat ΔpriB::dhfr recA269*::Tn*10*	AM2078 x P1.N3072 to Tc^r

AM2097	*ΔruvABC*::*cat ΔpriB*::*dhfr tnaA*::Tn*10 recF143*	AM2078 x P1.JC12334 to Tc^{r}
AM2106	*ΔpriB*::*dhfr tnaA*::Tn*10 recF143*	AM2077 x P1.JC12334 to Tc^{r}
AM2123	*ΔrecG*::*apra*	AB1157 x P1.N6052 to Apra^{r}
AM2124	*ΔruvABC*::*cat ΔrecG*::*apra*	N4454 x P1.N6052 to Apra^{r}
AM2131	*ΔruvABC*::*cat ΔpriB*::*dhfr ΔrecR*::*kan*	AM2078 x P1.AM1816 to Km^{r}
AM2132	*ΔruvABC*::*cat ΔpriB*::*dhfr ΔrecO*::*kan*	AM2078 x P1.AM1746 to Km^{r}
AM2133	*ΔruvABC*::*cat ΔpriB*::*dhfr recJ284*::*Tn*10	AM2078 x P1.N4934 to Tc^{r}
AM2134	*ΔruvABC*::*cat ΔpriB*::*dhfr ΔrecQ*::*kan*	AM2078 x P1.N5602 to Km^{r}
AM2138	*ΔpriB*::*dhfr ΔrecR*::*kan*	AM2077 x P1.AM1816 to Km^{r}
AM2139	*ΔpriB*::*dhfr ΔrecO*::*kan*	AM2077 x P1.AM1746 to Km^{r}
AM2140	*ΔpriB*::*dhfr recJ284*::*Tn*10	AM2077 x P1.N4934 to Tc^{r}
AM2141	*ΔpriB*::*dhfr ΔrecQ*::*kan*	AM2077 x P1.N5602 to Km^{r}
AM2142	*ΔpriB*::*dhfr recB268*::Tn*10*	AM2077 x P1.TRM308 to Tc^{r}
AM2147	*ΔruvABC*::*cat ΔrecR*::*kan*	N4454 x P1.AM1816 to Km^{r}
AM2148	*ΔruvABC*::*cat ΔrecO*::*kan*	N4454 x P1.AM1746 to Km^{r}
AM2149	*ΔruvABC*::*cat recJ284*::*Tn*10	N4454 x P1.N4934 to Tc^{r}
AM2160	*ΔrecR*::*kan*	AB1157 x P1.AM1816 to Km^{r}
AM2161	*ΔrecO*::*kan*	AB1157 x P1.AM1746 to Km^{r}
AM2213	*ΔpriB*::*dhfr arg*$^{+}$ *rpoB[G1260D]*	AM2077 x P1.AM2073 to Arg^{+}
N2446	*recJ284*::*Tn*10	(Ryder *et al.*, 1994)
N4361	*tnaA*::Tn*10 recF143*	AB1157 x P1.JC12334 to Tc^{r}
N4454	*ΔruvABC*::*cat*	(Jaktaji and Lloyd, 2003)
N5165	*sfiA11*	(Dri *et al.*, 1991)
N5208	*sfiA11*	(Trautinger *et al.*, 2005)
N5218	*sfiA11 ΔruvABC*::*cat*	N5208 x P1.N4884 to Cm^{r}
N7079	*ΔrecQ*::*kan*	AB1157 x P1.N6499 to Km^{r}
N7082	*ΔruvABC*::*cat ΔrecQ*::*kan*	N4454 x P1.N6499 to Km^{r}
N7911	*ΔpriB*::*dhfr zji-202*::Tn*10 dnaC809,820*	AM2077 x P1.N6424 to Tc^{r}
N7915	*ΔpriB*::*dhfr zji-202*::Tn*10 dnaC809,820 ΔruvABC*::*cat*	N7911 x P1.N4884 to Cm^{r}
N7918	*ΔpriB*::*dhfr zji-202*::Tn*10 dnaC809,820 ΔpriC303*::*kan*	N7911 x P1.N6424 to Km^{r}
N7926	*ΔpriB*::*dhfr zji-202*::Tn*10 dnaC809,820 ΔpriC303*::*kan ΔruvABC*::*cat*	N7918 x P1.N4884 to Cm^{r}
N7933	*ΔpriC303*::*kan*	AB1157 x P1.N6424 to Km^{r}
N7934	*ΔruvABC*::*cat ΔpriC303*::*kan*	N4454 x P1.N6424 to Km^{r}
N7936	*ΔpriB*::*dhfr malE*::Tn*10 lexA3*	AM2077 x P1.N5123 to Tc^{r}
N7938	*ΔpriB*::*dhfr malE*::Tn*10 lexA3 ΔruvABC*::*cat*	N7936 x P1.N4884 to Cm^{r}
N7940	*sfiA11 ΔruvABC*::*cat ΔpriB*::*dhfr*	N5218 x P1.AM2013 to Tm^{r}
N7944	*ΔruvABC*::*cat purE85*::Tn*10*	N4454 x P1.N3005 to Tc^{r}
N7945	*ΔruvABC*::*cat rus-2* (*orf-56*::IS*10*)	N7944 x P1.N4574 to Pur^{+}
N7946	*ΔruvABC*::*cat rus-2* (*orf-56*::IS*10*) *ΔpriB*::*dhfr*	N7945 x P1.AM2013 to Tm^{r}
N7947	*rpoB*[G1260D] *arg*$^{+}$	AB1157 x P1.AM2073 to Arg^{+}
N7948	*rpoB*[G1260D] *arg*$^{+}$ *ΔruvABC*::*cat*	N7947 x P1.N4884 to Cm^{r}
N7950	*rpoB*35 arg*$^{+}$	AB1157 x P1.N4884 to Arg^{+}
N7951	*rpoB*35 arg*$^{+}$ *ΔruvABC*::*cat*	N7950 x P1.N4884 to Cm^{r}
N7959	*ΔruvABC*::*cat rus-1* (*orf-151*::IS2)	MC^{r} derivative of N4454
N7962	*zjf-920*::Tn*10 rpsF292*	AM2077 x P1.N7078 to Tc^{r} (Tm^{s})

N7963	*ΔrecG*::*apra zjf-920*::Tn*10 rpsF292*	AM2089 x P1.N7078 to Tc^{r} (Tm^{s})
N7964	*rpoB*[G1260D] *arg*$^{+}$ *ΔruvABC*::*cat ΔpriB*::*dhfr*	N7948 x P1.AM2013 to Tm^{r}
N7975	*ΔruvABC*::*cat rus-1* (*orf-151*::IS2) *ΔpriB*::*dhfr*	N7959 x P1.AM2013 to Tm^{r}
N7985	*zjf-920*::Tn*10 rpsF292 ΔrecG*::*apra*	N7962 x P1.N6052 to Apra^{r}
N7986	*zjf-920*::Tn*10 rpsF292 ΔruvABC*::*cat*	N7962 x P1.N4884 to Cm^{r}
N7987	*ΔrecG*::*apra zjf-920*::Tn*10 rpsF292 ΔruvABC*::*cat*	N7963 x P1.N4884 to Cm^{r}
N8003	*rpoB**35 *arg*$^{+}$ *ΔpriB*::*dhfr*	N7950 x P1.AM2013 to Tm^{r}
N8004	*rpoB**35 *arg*$^{+}$ *ΔpriB*::*dhfr ΔruvABC*::*cat*	N8003 x P1.N4884 to Cm^{r}
N8035	*ΔruvABC*::*cat ΔpriB*::*dhfr recB268*::Tn*10*	AM2078 x P1.TRM308 to Tc^{r}
N8163	*ΔpriB*::*dhfr yheB*::*kan rpoA[S49T,S309P]*	AM2077 x P1.AM2204 to Km^{r}
N8164	*ΔpriB*::*dhfr yheB*::*kan rpoA[E273D]*	AM2077 x P1.AM2205 to Km^{r}
N8167	*ΔpriB*::*dhfr arg*$^{+}$ *rpoC[ΔK215-R220]*	AM2077 x P1.AM2059 to Arg^{+}
N8168	*ΔpriB*::*dhfr arg*$^{+}$ *rpoB[ΔD446-L448]*	AM2077 x P1.AM2063 to Arg^{+}
N8169	*ΔpriB*::*dhfr arg*$^{+}$ *rpoB[S1332L]*	AM2077 x P1.AM2069 to Arg^{+}
N8170	*ΔpriB*::*dhfr arg*$^{+}$ *rpoB[R452L]*	AM2077 x P1.AM2070 to Arg^{+}
N8172	*ΔpriB*::*dhfr yheR*::*kan rpoA[K298N] rpsL*$^{+}$	AM2077 x P1.AM2231 to Km^{r}
N8174	*ΔpriB*::*dhfr yheB*::*kan rpoA[S49T,S309P] ΔruvABC*::*cat*	N8163 x P1.N4884 to Cm^{r}
N8175	*ΔpriB*::*dhfr yheB*::*kan rpoA[E273D] ΔruvABC*::*cat*	N8164 x P1.N4884 to Cm^{r}
N8178	*ΔpriB*::*dhfr arg*$^{+}$ *rpoC[ΔK215-R220] ΔruvABC*::*cat*	N8167 x P1.N4884 to Cm^{r}
N8179	*ΔpriB*::*dhfr arg*$^{+}$ *rpoB[ΔD446-L448] ΔruvABC*::*cat*	N8168 x P1.N4884 to Cm^{r}
N8180	*ΔpriB*::*dhfr arg*$^{+}$ *rpoB[S1332L] ΔruvABC*::*cat*	N8169 x P1.N4884 to Cm^{r}
N8181	*ΔpriB*::*dhfr arg*$^{+}$ *rpoB[R452L] ΔruvABC*::*cat*	N8170x P1.N4884 to Cm^{r}
N8184	*ΔpriB*::*dhfr yheR*::*kan rpoA[K298N] rpsL[K43R]*	Selection for Str^{r} derivative of N8172
N8185	*ΔpriB*::*dhfr yheR*::*kan rpoA[K298N] rpsL[K43R] ΔruvABC*::*cat*	N8184 x P1.N4884 to Cm^{r}
N8186	*ΔpriB*::*dhfr yheB*::*kan rpoA{L253R]*	AM2077 x P1.AM2210 to Km^{r}
N8187	*ΔpriB*::*dhfr yheB*::*kan rpoA{L253R] ΔruvABC*::*cat*	N8186 x P1.N4884 to Cm^{r}

MG1655 and derivatives

MG1655	F^{-} *rph-1*	(Bachmann, 1996)
ACE712	*metB1 argE86*::Tn*10 ΔrecG*::*apra yjcB*::*kan rpsF292*	MC resistant derivative of JJ1643
ACE730	*metB1 ΔrecG*::*apra yjcB*::*kan rpsF292*	Arg^{+} (Tc^{s}) derivative of ACE712
ACE732	*metB1 ΔrecG*::*apra rpsF292 malE*::Tn*10*	ACE730 x P1.N2231 to Tc^{r} (Km^{s})
ACE734	*metB1 ΔrecG*::*apra malE*::Tn*10 mutL*::*kan*	ACE732 x P1.MG1655::EZ*kan* pool to Km^{r} (MC^{s})
ACE739	*metB1 ΔrecG*::*apra rpsF292 malE*::Tn*10 mutL*::*kan*	ACE732 x P1.ACE734 to Km^{r}
AM1417	*ΔpyrE*::*dhfr*	(Zhang *et al.*, 2010a)
AM1746	*ΔrecO*::*kan*	(Rudolph *et al.*, 2008)
AM1771	*Δdam*::*dhfr*	This work
AM1816	*ΔrecR*::*kan*	This work
AM2013	*ΔpriB*::*dhfr*	This work
AM2017	*ΔlacIZYA ΔpriB*::*dhfr*	TB28 x P1.AM2013
AM2018	*ΔlacIZYA ΔrecG*::*apra ΔpriB*::*dhfr*	N6537 x P1.AM2013
AM2023	*ΔlacIZYA ΔrecG*::*apra zjf-920*::Tn*10 rpsF292*	N6576 x P1.N7078 to Tc^{r}
AM2024	*ΔrpsF*::*cat*	This work
AM2030	*ΔlacIZYA ΔrecG*::*apra ΔrpsF*::*cat*	N6576 x P1.AM2024 to Cm^{r}
AM2032	*ΔlacIZYA ΔrpsF*::*cat*	TB28 x P1.AM2024 to Cm^{r}

AM2038	*ΔlacIZYA* pAM421 (*priC*$^+$ *lac*$^+$) *ΔpriC303*::*kan ΔpriB*::*dhfr*	N6813 x P1.AM2013 to Tm^r
AM2043	*ΔlacIZYA ΔpriB*::*dhfr* pAM421 (*priC*$^+$ *lac*$^+$)	AM2017 x pAM421 to Ap^r
AM2055	*ΔlacIZYA ΔrecG*::*apra zjf-920*::Tn*10 ΔpriB202*	N6576 x P1.AM2054 to Tc^r
AM2057	*ΔlacIZYA ΔrecG*::*apra zjf-920*::Tn*10 ΔpriB202 supGB1A*[c]	MC^r derivative of AM2055
AM2058	*ΔlacIZYA ΔrecG*::*apra zjf-920*::Tn*10 ΔpriB202 supGB1B*[c]	MC^r derivative of AM2055
AM2059	*ΔlacIZYA ΔrecG*::*apra zjf-920*::Tn*10 ΔpriB202 rpoC[ΔK215-R220]*	MC^r derivative of AM2055
AM2060	*ΔlacIZYA ΔrecG*::*apra zjf-920*::Tn*10 ΔpriB202 rpoB*[S1332L]	MC^r derivative of AM2055
AM2061	*ΔlacIZYA ΔrecG*::*apra zjf-920*::Tn*10 ΔpriB202 supGB1C*[c]	MC^r derivative of AM2055
AM2062	*ΔlacIZYA ΔrecG*::*apra zjf-920*::Tn*10 ΔpriB202 supGB1D*[c]	MC^r derivative of AM2055
AM2063	*ΔlacIZYA ΔrecG*::*apra zjf-920*::Tn*10 ΔpriB202 rpoB[ΔD446-L448]*	MC^r derivative of AM2055
AM2064	*ΔlacIZYA ΔrecG*::*apra zjf-920*::Tn*10 ΔpriB202 rpoA[P293L]*	MC^r derivative of AM2055
AM2065	*ΔlacIZYA ΔrecG*::*apra zjf-920*::Tn*10 ΔpriB202 supGB1G*[c]	MC^r derivative of AM2055
AM2066	*ΔlacIZYA ΔrecG*::*apra zjf-920*::Tn*10 ΔpriB202 rpoA[P293L]*	MC^r derivative of AM2055
AM2067	*ΔlacIZYA ΔrecG*::*apra zjf-920*::Tn*10 ΔpriB202 rpoA[L253R]*	MC^r derivative of AM2055
AM2069	*ΔlacIZYA ΔrecG*::*apra zjf-920*::Tn*10 ΔpriB202 rpoB*[S1332L]	MC^r derivative of AM2055
AM2070	*ΔlacIZYA ΔrecG*::*apra zjf-920*::Tn*10 ΔpriB202 rpoB*[R452L]	MC^r derivative of AM2055
AM2071	*ΔlacIZYA ΔrecG*::*apra zjf-920*::Tn*10 ΔpriB202 rpoA[S49T, S309P]*	MC^r derivative of AM2055
AM2072	*ΔlacIZYA ΔrecG*::*apra zjf-920*::Tn*10 ΔpriB202 rpoA[P293L]*	MC^r derivative of AM2055
AM2073	*ΔlacIZYA ΔrecG*::*apra zjf-920*::Tn*10 ΔpriB202 rpoB*[G1260D]	MC^r derivative of AM2055
AM2074	*ΔlacIZYA ΔrecG*::*apra zjf-920*::Tn*10 ΔpriB202 rpoA[E273D]*	MC^r derivative of AM2055
AM2075	*ΔlacIZYA ΔrecG*::*apra zjf-920*::Tn*10 ΔpriB202 rpoA[P293L]*	MC^r derivative of AM2055
AM2129	*rpoB*35 ΔrecG*::*apra*	N4849 x P1.N6052 to Apra^r
AM2130	*rpoB*35 ΔpriB*::*dhfr*	N4849 x P1.AM2013 to Tm^r
AM2135	*rpoB*35 ΔrecG*::*apra ΔpriB*::*dhfr*	AM2129 x P1.AM2013 to Tm^r
AM2146	*ΔlacIZYA ΔpriB*::*dhfr ΔruvABC*::*cat*	AM2017 x P1.N4884 to Cm^r
AM2153	*ΔlacIZYA zji-202*::Tn*10 dnaC809,820 ΔpriB*::*dhfr*	N6485 x P1.AM2013 to Tm^r
AM2155	*ΔlacIZYA argE86*::Tn*10*	TB28 x P1.N4837 to Tc^r
AM2156	*ΔlacIZYA argE86*::Tn*10 rpoB[I572F]*	High rifampicin resistant derivative of AM2155
AM2157	*ΔlacIZYA rpoB[S1332L]*	AM2156 x P1.AM2069 to Arg$^+$
AM2158	*ΔlacIZYA rpoB[G1260D]*	AM2156 x P1.AM2073 to Arg$^+$
AM2165	*ΔlacIZYA ΔrecG*::*apra zjf-920*::Tn*10 ΔpriB202 yheB*::*kan*	AM2064 x P1.MG1655::EZ*kan* pool to Km^r
AM2166	*ΔlacIZYA ΔrecG*::*apra zjf-920*::Tn*10 ΔpriB202 yheB*::*kan*	AM2064 x P1.MG1655::EZ*kan* pool to Km^r
AM2167	*ΔlacIZYA ΔrecG*::*apra zjf-920*::Tn*10 ΔpriB202 yheR*::*kan*	AM2064 x P1.MG1655::EZ*kan* pool to Km^r
AM2173	*ΔlacIZYA ΔrecG*::*apra zjf-920*::Tn*10 ΔpriB202 yheB*::*kan rpoA[P293L]*	MC^r derivative of AM2166
AM2174	*ΔlacIZYA ΔrecG*::*apra zjf-920*::Tn*10 ΔpriB202 yheR*::*kan rpoA[K298N]*	MC^r derivative of AM2167
AM2177	*ΔlacIZYA yheB*::*kan*	TB28 x P1.AM2173 to Km^r
AM2178	*ΔlacIZYA yheB*::*kan rpoA[P293L]*	TB28 x P1.AM2173 to Km^r
AM2182	*ΔlacIZYA rpoB[S1332L] ΔrecG*::*apra*	AM2157 x P1.N6052 to Apra^r
AM2183	*ΔlacIZYA rpoB[S1332L] ΔpriB*::*dhfr*	AM2157 x P1.AM2013 to Tm^r

AM2184	*ΔlacIZYA rpoB[G1260D] ΔrecG::apra*	AM2158 x P1.N6052 to Apra^r
AM2185	*ΔlacIZYA rpoB[G1260D] ΔpriB::dhfr*	AM2158 x P1.AM2013 to Tm^r
AM2190	*ΔlacIZYA rpoB[S1332L] ΔpriB::dhfr ΔrecG::apra*	AM2183 x P1.N6052 to Apra^r
AM2191	*ΔlacIZYA ΔrecG::apra zjf-920::*Tn*10 ΔpriB202 yheB::kan rpoA[P293L]*	MC^r derivative of AM2165
AM2193	*ΔlacIZYA rpoB[G1260D] ΔpriB::dhfr ΔrecG::apra*	AM2185 x P1.N6052 to Apra^r
AM2196	*ΔlacIZYA yheB::kan*	TB28 x P1.AM2191 to Km^r
AM2197	*ΔlacIZYA yheB::kan rpoA[P293L]*	TB28 x P1.AM2191to Km^r
AM2198	*ΔlacIZYA yheB::kan rpoA[P293L] ΔrecG::apra*	AM2197 x P1.N6052 to Apra^r
AM2199	*ΔlacIZYA yheB::kan rpoA[P293L] ΔpriB::dhfr*	AM2197 x P1.AM2013 to Tm^r
AM2200	*ΔlacIZYA yheB::kan rpoA[P293L] ΔpriB::dhfr ΔrecG::apra*	AM2199 x P1.N6052 to Apra^r
AM2204	*ΔlacIZYA ΔrecG::apra zjf-920::*Tn*10 ΔpriB202 rpoA[S49T, S309P] yheB::kan*	AM2071 x P1.AM2177 to Km^r
AM2205	*ΔlacIZYA ΔrecG::apra zjf-920::*Tn*10 ΔpriB202 rpoA[E273D] yheB::kan*	AM2074 x P1.AM2177 to Km^r
AM2208	*ΔlacIZYA yheB::kan rpoA[S49T, S309P]*	TB28 x P1.AM2204 to Km^r
AM2209	*ΔlacIZYA yheB::kan rpoA[E273D]*	TB28 x P1.AM2205 to Km^r
AM2210	*ΔlacIZYA ΔrecG::apra zjf-920::*Tn*10 ΔpriB202 yheB::kan rpoA[L253R]*	AM2067 x P1.AM2177
AM2231	*ΔlacIZYA yheR::kan rpoA[K298N]*	TB28 x P1.AM2174 to Km^r
AM2232	*ΔlacIZYA yheR::kan rpoA[K298N] ΔpriB::dhfr*	AM2231 x P1.AM2013 to Tm^r
AM2233	*ΔlacIZYA yheR::kan rpoA[K298N] ΔrecG::apra*	AM2231 x P1.N6052 to Apra^r
AM2234	*ΔlacIZYA yheR::kan rpoA[K298N] ΔpriB::dhfr ΔrecG::apra*	AM2232 x P1.N6052 to Apra^r
AM2328	*ΔlacIZYA ΔrecG::apra zjf-920::*Tn*10 rpsF292* pJJ100 (*recG*$^+$ *lac*$^+$)	AM2023 x pJJ100 to Ap^r
AM2329	*ΔlacIZYA ΔrecG::apra zjf-920::*Tn*10 rpsF292* pJJ100 (*recG*$^+$ *lac*$^+$) *Δdam::dhfr*	AM2328 x P1.AM1771 to Tm^r
AM2330	*ΔlacIZYA ΔrecG::apra zjf-920::*Tn*10 rpsF292* pJJ100 (*recG*$^+$ *lac*$^+$) *polA::kan*	AM2328 x P1.JJ1038 to Km^r
AM2333	*ΔlacIZYA polA::kan* pAM475 (*polA*$^+$ *lac*$^+$) *ΔpriB::dhfr*	N7228 x P1.AM2013 to Tm^r
AM2343	*ΔlacIZYA rpoB[G1260D] ΔpriB::dhfr* pAM475 (*polA*$^+$ *lac*$^+$)	AM2185 x pAM475 to Ap^r
AM2346	*ΔlacIZYA rpoB[G1260D] ΔpriB::dhfr* pAM475 (*polA*$^+$ *lac*$^+$) *polA::kan*	AM2343 x P1.JJ1038 to Km^r
AM2379	*ΔlacIZYA rpoB[ΔD446-L448]*	AM2156 x P1.AM2063 to Arg$^+$
AM2380	*ΔlacIZYA rpoB[R452L]*	AM2156 x P1.AM2070 to Arg$^+$
AM2381	*ΔlacIZYA rpoC[ΔK215-R220]*	AM2156 x P1.AM2059 to Arg$^+$
AM2410	*ΔlacIZYA ΔrecG::apra ΔrpsF::cat* pGB061(*rpsF*$^+$)	AM2030 x pGB061 to Ap^r
AM2411	*ΔlacIZYA ΔrpsF::cat* pGB061(*rpsF*$^+$)	AM2032 x pGB061 to Ap^r
AM2412	*ΔlacIZYA ΔrecG::apra ΔrpsF::cat* pTRc99A	AM2030 x pTRc99A to Ap^r
AM2413	*ΔlacIZYA ΔrpsF::cat* pTRc99A	AM2032 x pTRc99A to Ap^r
JJ1038	*polA::kan*	(Zhang et al., 2010a)
JJ1119	*ΔlacIZYA ΔrecG::apra* pJJ100 (*recG*$^+$ *lac*$^+$)	(Zhang et al., 2010a)
JJ1122	*ΔlacIZYA ΔrecG::apra dam1::kan* pJJ100 (*recG*$^+$ *lac*$^+$)	(Zhang et al., 2010a)
JJ1123	*ΔlacIZYA ΔrecG::apra polA::kan* pJJ100 (*recG*$^+$ *lac*$^+$)	(Zhang et al., 2010a)
JJ1160	*ΔlacIZYA polA::kan*	(Zhang et al., 2010a)
JJ1489	*ΔlacIZYA ΔcysG::apra ssb[A130G]*	(Zhang et al., 2010a)
JJ1495	*ΔlacIZYA ΔcysG::apra yjcB::kan*	(Zhang et al., 2010a)
JJ1506	*ΔlacIZYA ΔcysG::apra yjcB::kan*	JJ1489 x P1.JJ1495 to Km^r

JJ1643	*metB1 argE86*::Tn*10 ΔrecG*::*apra yjcB*::*kan*	N6769 x P1.JJ1506 to Km^r
N4560	*ΔrecG265*::*cat*	(Mahdi et al., 2006)
N4574	*relA1 ΔspoT207*::*cat rpoB*35 eda-51*::Tn*10 ΔruvAC65 rus-2* (*orf-56*::IS*10*)	(Mahdi et al., 2006)
N4583	*ΔruvABC*::*cat*	(Jaktaji and Lloyd, 2003)
N4837	*argE86*::Tn*10*	(Jaktaji and Lloyd, 2003)
N4849	*rpoB*35 = rpoB[H1244Q]*	(Jaktaji and Lloyd, 2003)
N4853	*ΔruvABC*::*cat argE86*::Tn*10*	N4583 x P1.N4837 to Tc^r
N4884	*rpoB*35 ΔruvABC*::*cat*	(Mahdi et al., 2006)
N4934	*recJ284*::Tn*10*	(Mahdi et al., 2006)
N5123	*malE*::Tn*10 lexA3*	(Trautinger *et al.*, 2005)
N5187	*pyrD sfiA100*::*kan*	(Rudolph *et al.*, 2009)
N5209	*sfiA11*	N5187 x P1.N5165 to Pyr$^+$
N5225	*sfiA11 ΔrecG265*::*cat*	N5209 x P1. N4452 to Cm^r
N5602	*ΔrecQ*::*kan*	(Mahdi et al., 2006)
N5675	*ΔpriC303*::*kan*	MG1655 x P1.JJC1405 to Km^r
N6052	*ΔrecG*::*apra*	(Mahdi et al., 2006)
N6485	*ΔlacIZYA zji-202*::Tn*10 dnaC809,820*	TB28 x P1.N6424 to Tc^r
N6490	*ΔlacIZYA zji-202*::Tn*10 dnaC809,820 ΔpriC303*::*kan*	N6485 x P1.N6424 to Km^r
N6499	*priA300 ΔlacIZYA ΔrecQ*::*kan ΔuvrD*::*dhfr*	(Guy *et al.*, 2009)
N6537	*ΔlacIZYA ΔrecG*::*apra*	Plasmid-free segregant of JJ1119
N6576	*ΔlacIZYA ΔrecG*::*apra*	(Zhang et al., 2010a)
N6629	*ΔlacIZYA ΔrecG*::*apra ΔruvABC*::*cat*	Plasmid-free segregant of N6628
N6754	*metB1*	N4837 x P1.KL227 to Arg$^+$ (Met$^-$)
N6755	*metB1 argE86*::Tn*10*	N6754 x P1.N4837 to Tc^r
N6769	*metB1 argE86*::Tn*10 ΔrecG*::*apra*	N6755 x P1.N6052 to Apra^r
N6806	*ΔlacIZYA* pAM421 (*priC$^+$ lac$^+$*)	TB28 x pAM421 to Ap^r
N6813	*ΔlacIZYA* pAM421 (*priC$^+$ lac$^+$*) *ΔpriC303*::*kan*	N6806 x P1.N6424 to Km^r
N7039	*ΔlacIZYA ΔrecG*::*apra mutL*::*kan rpsF292*	N6576 x P1.ACE739 to Km^r
N7073	*ΔlacIZYA ΔrecG*::*apra mutL*::*kan rpsF292 zjf-920*::Tn*10*	N7039 x P1.CGSC7484 to Tc^r
N7078	*ΔlacIZYA ΔrecG*::*apra zjf-920*::Tn*10 rpsF292*	N6576 x P1.N7073 to Tc^r
N7088	*ΔlacIZYA zjf-920*::Tn*10 rpsF292 ΔpyrE*::*dhfr*	N7078 x P1.AM1417 to Tm^r
N7090	*ΔlacIZYA zjf-920*::Tn*10 rpsF292*	N7088 x P1.W3110 to Pyr$^+$ (Tm^s)
N7091	*ΔlacIZYA ΔrecG*::*apra zjf-920*::Tn*10 rpsF292 ΔruvABC*::*cat*	N7078 x P1.N4884 to Cm^r
N7093	*ΔlacIZYA zjf-920*::Tn*10 rpsF292 ΔruvABC*::*cat*	N7090 x P1.N4884 to Cm^r
N7228	*ΔlacIZYA polA*::*kan* pAM475 (*polA$^+$ lac$^+$*)	JJ1160 x pAM475 to Ap^r
N7726	*ΔruvABC*::*cat rpoB[G1260D]*	N4853 x P1.AM2073 to Arg$^+$
N7730	*ΔlacIZYA rpoB[S1332L] ΔruvABC*::*cat*	AM2157 x P1.N4884 to Cm^r
N7900	*ΔlacIZYA* pAM421 (*priC$^+$ lac$^+$*) *ΔpriC303*::*kan zji-202*::Tn*10 dnaC809,820*	N6813 x P1.N6424 to Tc^r
N7908	*ΔlacIZYA* pAM421 (*priC$^+$ lac$^+$*) *ΔpriC303*::*kan zji-202*::Tn*10 dnaC809,820 ΔpriB*::*dhfr*	N7900 x P1.AM2013 to Tm^r
N7941	*sfiA11 ΔpriB*::*dhfr*	N5209 x P1.AM2013 to Tm^r
N7943	*sfiA11 ΔrecG265*::*cat ΔpriB*::*dhfr*	N5225 x P1.AM2013 to Tm^r
N7949	*ΔruvABC*::*cat rpoB[G1260D] ΔpriB*::*dhfr*	N7726 x P1.AM2013 to Tm^r
N7967	*ΔpriC303*::*kan ΔruvABC*::*cat*	N5675 x P1.N4884 to Cm^r
N7968	*ΔlacIZYA ΔpriC303*::*kan zji-202*::Tn*10 dnaC809,820 ΔpriB*::*dhfr*	Plasmid free segregant of N7908

N7969	*ΔlacIZYA ΔpriC303::kan zji-202::*Tn*10 dnaC809,820 ΔpriB::dhfr ΔruvABC::cat*	N7968 x P1.N4884 to Cm^{r}
N7978	*ΔlacIZYA zji-202::*Tn*10 dnaC809,820 ΔruvABC::cat*	N6485 x P1.N4884 to Cm^{r}
N8018	*ΔlacIZYA polA::kan ΔpriB::dhfr*	Plasmid free segregant of AM2333 on minimal agar
N8029	*ΔlacIZYA rpoB[G1260D] ΔpriB::dhfr polA::kan*	Plasmid free segregant of AM2346 on minimal agar
N8036	*ΔlacIZYA zji-202::*Tn*10 dnaC809,820 ΔpriB::dhfr ΔruvABC::cat*	AM2153 x P1.N4884 to Cm^{r}
N8037	*ΔlacIZYA yheB::kan rpoA[E273D] ΔruvABC::cat*	AM2209 x P1.N4884 to Cm^{r}
N8043	*ΔlacIZYA yheB::kan rpoA[P293L] ΔruvABC::cat*	AM2178 x P1.N4884 to Cm^{r}
N8044	*ΔlacIZYA yheB::kan rpoA[S49T, S309P] ΔruvABC::cat*	AM2208 x P1.N4884 to Cm^{r}
N8045	*ΔlacIZYA yheR::kan rpoA[K298N] ΔruvABC::cat*	AM2231 x P1.N4884 to Cm^{r}
N8056	*ΔlacIZYA rpoB[ΔD446-L448] ΔruvABC::cat*	AM2379 x P1.N4884 to Cm^{r}
N8057	*ΔlacIZYA rpoB[R452L] ΔruvABC::cat*	AM2380 x P1. N4884 to Cm^{r}
N8058	*ΔlacIZYA rpoC[ΔK215-R220] ΔruvABC::cat*	AM2381 x P1. N4884 to Cm^{r}
N8059	*ΔlacIZYA rpoA[E273D]*	AM2178 x P1.AM2074 to Met^{+} (Km^{s})[d]
N8069	*ΔlacIZYA rpoA[E273D] ΔrecG::apra*	N8059 x P1.N6052 to Apra^{r}
N8070	*ΔlacIZYA rpoA[E273D]*	N8059 x P1. N4884 to Cm^{r}
N8072	*ΔlacIZYA rpoA[E273D] ΔpriB::dhfr*	N8059 x P1.AM2013 to Tm^{r}
N8077	*ΔlacIZYA rpoA[E273D] ΔpriB::dhfr ΔrecG::apra*	N8072 x P1.N6052 to Apra^{r}
N8078	*ΔlacIZYA rpoC[ΔK215-R220] ΔrecG::apra*	AM2381 x P1.N6052 to Apra^{r}
N8079	*ΔlacIZYA rpoC[ΔK215-R220] ΔpriB::dhfr*	AM2381 x P1.AM2013 to Tm^{r}
N8080	*ΔlacIZYA rpoC[ΔK215-R220] ΔpriB::dhfr ΔrecG::apra*	N8079 x P1.N6052 to Apra^{r}
N8086	*ΔlacIZYA rpoA[S49T, S309P]*	AM2178 x P1.AM2071 to Met^{+} (Km^{s})[d]
N8087	*ΔlacIZYA rpoA[S49T, S309P] ΔrecG::apra*	N8086 x P1.N6052 to Apra^{r}
N8088	*ΔlacIZYA rpoA[S49T, S309P] ΔpriB::dhfr*	N8086 x P1.AM2013 to Tm^{r}
N8089	*ΔlacIZYA rpoA[S49T, S309P] ΔpriB::dhfr ΔrecG::apra*	N8088 x P1.N6052 to Apra^{r}
N8090	*ΔlacIZYA rpoB[ΔD446-L448] ΔrecG::apra*	AM2379 x P1.N6052 to Apra^{r}
N8091	*ΔlacIZYA rpoB[ΔD446-L448] ΔpriB::dhfr*	AM2379 x P1.AM2017 to Tm^{r}
N8092	*ΔlacIZYA rpoB[ΔD446-L448] ΔpriB::dhfr ΔrecG::apra*	N8091 x P1.N6052 to Apra^{r}
N8094	*ΔlacIZYA yheB::*kan *rpoA[L253R]*	TB28 x P1.AM2210 to Km^{r}
N8095	*ΔlacIZYA yheB::*kan *rpoA[L253R] ΔrecG::apra*	N8094 x P1.N6052 to Apra^{r}
N8096	*ΔlacIZYA yheB::*kan *rpoA[L253R] ΔpriB::dhfr*	N8094 x P1.AM2017 to Tm^{r}
N8097	*ΔlacIZYA yheB::*kan *rpoA[L253R]*	N8094 x P1. N4884 to Cm^{r}
N8101	*ΔlacIZYA rpoB[R452L] ΔrecG::apra*	AM2380 x P1.N6052 to Apra^{r}
N8102	*ΔlacIZYA rpoB[R452L] ΔpriB::dhfr*	AM2380 x P1.AM2017 to Tm^{r}
N8104	*ΔlacIZYA rpoA[L253R] ΔpriB::dhfr ΔrecG::apra*	N8096 x P1.N6052 to Apra^{r}
N8106	*ΔlacIZYA rpoB[R452L] ΔpriB::dhfr ΔrecG::apra*	N8102 x P1.N6052 to Apra^{r}
N8111	*ΔlacIZYA rpoA[E273D] ΔpriB::dhfr* pAM475(*polA*$^{+}$ *lac*$^{+}$)	N8072 x pAM475 to Ap^{r}
N8112	*ΔlacIZYA rpoC[ΔK215-R220] ΔpriB::dhfr* pAM475(*polA*$^{+}$ *lac*$^{+}$)	N8079 x pAM475 to Ap^{r}
N8116	*ΔlacIZYA rpoA[E273D] ΔpriB::dhfr* pAM475(*polA*$^{+}$ *lac*$^{+}$) *polA::kan*	N8111 x P1.JJ1038 to Km^{r}
N8117	*ΔlacIZYA rpoC[ΔK215-R220] ΔpriB::dhfr* pAM475(*polA*$^{+}$ *lac*$^{+}$) *polA::kan*	N8112 x P1.JJ1038 to Km^{r}
N8136	*ΔlacIZYA rpoA[E273D] ΔpriB::dhfr ΔruvABC::cat*	N8072 x P1.N4884 to Cm^{r}

N8137	*ΔlacIZYA rpoC[ΔK215-R220] ΔpriB::dhfr ΔruvABC::cat*	N8079 x P1.N4884 to Cm^r
N8138	*ΔlacIZYA rpoA[S49T, S309P] ΔpriB::dhfr ΔruvABC::cat*	N8088 x P1.N4884 to Cm^r
N8139	*ΔlacIZYA rpoB[ΔD446-L448] ΔpriB::dhfr ΔruvABC::cat*	N8091 x P1.N4884 to Cm^r
N8140	*ΔlacIZYA yheB::*kan *rpoA[L253R] ΔpriB::dhfr ΔruvABC::cat*	N8096 x P1.N4884 to Cm^r
N8141	*ΔlacIZYA rpoB[R452L] ΔpriB::dhfr ΔruvABC::cat*	N8102 x P1.N4884 to Cm^r
N8142	*ΔlacIZYA zji-202::*Tn*10 dnaC809,820 ΔpriB::dhfr* pAM475 (*polA*$^+$ *lac*$^+$)	AM2153 x pAM475 to Ap^r
N8145	*ΔlacIZYA zji-202::*Tn*10 dnaC809,820 ΔpriB::dhfr* pAM475 (*polA*$^+$ *lac*$^+$) *polA::kan*	N8142 x P1.JJ1038 to Km^r
N8159	*ΔlacIZYA rpoB[S1332L] ΔpriB::dhfr ΔruvABC::cat*	AM2183 x P1.N4884 to Cm^r
N8160	*ΔlacIZYA yheB::kan rpoA[P293L] ΔpriB::dhfr ΔruvABC::cat*	AM2199 x P1.N4884 to Cm^r
N8161	*ΔlacIZYA yheR::kan rpoA[K298N] ΔpriB::dhfr ΔruvABC::cat*	AM2232 x P1.N4884 to Cm^r
TB28	*ΔlacIZYA<>frt*[d]	(Bernhardt and de Boer, 2003)

[a]Only the relevant additional genotype of the AB1157 and MG1655 derivatives are shown. The *ΔlacIZYA* allele listed = *ΔlacIZYA<>frt* (Bernhardt and de Boer, 2003).

[b]CGSC = Coli Genetic Stock Center.

[c]The *supGB* alleles in these strains are unidentified suppressors of the mitomycin C-sensitivity of AM2055.

[d]The *rpoA[P293L]* allele confers a requirement for methionine for growth in minimal salts media, i.e. confers a Met$^-$ phenotype. Selecting Met$^+$ allows for *rpoA[P293L]* to be replaced with the donor *rpoA* allele.

Supporting Results

Identification of rpsF292 as a suppressor of recG.

Samples from cultures of strain JJ1643 (*ΔrecG metB1 argE*::Tn*10 yjcB*::*kan*) were spread on LB agar plates supplemented with mitomycin C at a final concentration of 0.5 μg/m and incubated at 37°C. Colonies of resistant derivatives (suppressors) were clearly visible within 48 h. P1 transductions revealed linkage of the suppressor to *metB* (~90%) and *argE* (~50%) in nearly all of the independent isolates examined, indicating that they most likely carried mutations in *priA*. Previous studies demonstrated that mutations reducing the helicase activity of PriA alleviate the mitomycin C sensitivity of a *recG* mutant (Al-Deib *et al.*, 1996, Zhang *et al.*, 2010b). However, the suppressor in one isolate with particularly strong resistance to mitomycin C revealed no linkage to the *metB* or *argE* markers in this strain (Table S1, ACE712). There was also no linkage to the *yjcB*::*kan* marker located very close to the *ssb* locus, thus eliminating the possibility that the suppressor was an *ssb* substitution of the type reported by Zhang et al (2010b).

To facilitate identification of the suppressor in strain ACE712, the *argE*::Tn*10* marker was first eliminated by selecting spontaneous Arg^+ clones on glucose minimal salts agar supplemented with methionine and the *yjcB*::*kan* marker then eliminated by introducing the linked *malE*::Tn*10*, screening for Tc^r Km^s clones. The resulting construct remained highly resistant to mitomycin C, indicating that it still carried the suppressor (Table S1, ACE732). This strain was then transduced with P1 phage grown on pools of cells carrying random *kan* insertions in the chromosome generated in strain MG1655 using the EZ-Tn5 <kan-2> Tnp Transposome system (Epicentre Technologies). The Km^r transductants were screened for those that were also sensitive to mitomycin C on the basis that such a clone would carry a *kan* insertion linked to the wild type allele of the suppressor locus. One candidate was identified and shown by PCR sequencing to carry an insertion in *mutL* at minute 94.7 of the genetic map (Fig. 1A; Table 1, ACE734). P1 phage from this clone was used to transduce the *ΔrecG metB1 malE*::Tn*10* suppressor strain ACE732 to Km^r. This time, 30% of the transductants tested proved sensitive to mitomycin C, i.e. had lost the suppressor. P1 grown on a transductant retaining the suppressor (ACE739) was used to transduce strain N6576 (*ΔrecG ΔlacIZYA*) to Km^r. 30% of the clones analysed proved resistant to mitomycin, i.e. had inherited the suppressor. One such isolate, labelled N7039, was then transduced to Tc^r with a P1 phage stock from strain CGSC7484, which carries a *zjf920*::Tn*10* insertion at minute 95.76 (Fig.1A). A transductant retaining both the suppressor and the linked *mutL*::*kan* marker was retained as strain N7073 (*ΔrecG ΔlacIZYA mutL*::*kan zjf920*::Tn*10*). Phage P1 from N7073 was then used to transduce N6576 (*ΔrecG ΔlacIZYA*) to Tc^r and the transductants were analysed for inheritance of the unselected *mutL* and the suppressor. The analysis indicated that the suppressor was located mid-way between *mutL* and *zjf920*, in the vicinity of *priB* at minute 95.3 (Fig. 1A; data not shown). PCR sequencing of this region in strain N7039 revealed no change in *priB*. However, an A to T transition was detected at position 292 in the upstream *rpsF* gene, converting the GAA codon for Glu98 to a TAA nonsense codon. This mutation was designated as *rpsF292* (Fig. 1A).

Supporting References

Al-Deib, A.A., Mahdi, A.A. and Lloyd, R.G. (1996) Modulation of recombination and DNA repair by the RecG and PriA helicases of *Escherichia coli* K-12. *J. Bacteriol.* **178**: 6782-6789.

Bachmann, B.J. (1996) Derivations and genotypes of some mutant derivatives of *Escherichia coli* K-12. In: *Escherichia coli* and *Salmonella* Cellular and Molecular Biology, (Second Edition). Neidhardt, F.C., Curtiss III,

R., Ingraham, J.L., Lin, E.C.C., Low, K.B., Magasanik, B., Reznikoff, W.S., Riley, M., Schaechter, M. and Umbarger, H.E. (eds). Washington, D.C.: ASM Press, pp. 2460-2488.

Bernhardt, T.G. and de Boer, P.A. (2003) The *Escherichia coli* amidase AmiC is a periplasmic septal ring component exported via the twin-arginine transport pathway. *Mol Microbiol* **48**: 1171-1182.

Dri, A.M., Rouviere-Yaniv, J. and Moreau, P.L. (1991) Inhibition of cell division in hupA hupB mutant bacteria lacking HU protein. *J Bacteriol* **173**: 2852-2863.

Guy, C.P., Atkinson, J., Gupta, M.K., Mahdi, A.A., Gwynn, E.J., Rudolph, C.J., Moon, P.B., van Knippenberg, I.C., Cadman, C.J., Dillingham, M.S., Lloyd, R.G. and McGlynn, P. (2009) Rep provides a second motor at the replisome to promote duplication of protein-bound DNA. *Mol Cell* **36**: 654-666.

Jaktaji, R.P. and Lloyd, R.G. (2003) PriA supports two distinct pathways for replication restart in UV-irradiated *Escherichia coli* cells. *Mol. Microbiol.* **47**: 1091-1100.

Lloyd, R.G. and Buckman, C. (1985) Identification and genetic analysis of *sbcC* mutations in commonly used *recBC sbcB* strains of *Escherichia coli* K-12. *J. Bacteriol.* **164**: 836-844.

Lloyd, R.G., Buckman, C. and Benson, F.E. (1987) Genetic analysis of conjugational recombination in *Escherichia coli* K-12 strains deficient in RecBCD enzyme. *J. Gen. Microbiol.* **133**: 2531-2538.

Mahdi, A.A., Buckman, C., Harris, L. and Lloyd, R.G. (2006) Rep and PriA helicase activities prevent RecA from provoking unnecessary recombination during replication fork repair. *Genes Dev.* **20**: 2135-2147.

Meddows, T.R., Savory, A.P. and Lloyd, R.G. (2004) RecG helicase promotes DNA double-strand break repair. *Mol Microbiol* **52**: 119-132.

Picksley, S.M., Lloyd, R.G. and Buckman, C. (1984) Genetic analysis and regulation of inducible recombination in *Escherichia coli* K-12. *Cold Spring Harbor Symp. Quant. Biol.* **49**: 469-474.

Rudolph, C.J., Mahdi, A.A., Upton, A.L. and Lloyd, R.G. (2010) RecG protein and single-strand DNA exonucleases avoid cell lethality associated with PriA helicase activity in *Escherichia coli. Genetics* **186**: 473-492.

Rudolph, C.J., Upton, A.L., Harris, L. and Lloyd, R.G. (2009) Pathological replication in cells lacking RecG DNA translocase. *Mol Microbiol* **73**: 352-366.

Rudolph, C.J., Upton, A.L. and Lloyd, R.G. (2008) Maintaining replication fork integrity in UV-irradiated *Escherichia coli* cells. *DNA Repair (Amst)* **7**: 1589-1602.

Ryder, L., Whitby, M.C. and Lloyd, R.G. (1994) Mutation of *recF, recJ, recO, recQ,* or *recR* improves Hfr recombination in resolvase-deficient *ruv recG* strains of *Escherichia coli. J. Bacteriol.* **176**: 1570-1577.

Trautinger, B.W., Jaktaji, R.P., Rusakova, E. and Lloyd, R.G. (2005) RNA polymerase modulators and DNA repair activities resolve conflicts between DNA replication and transcription. *Mol. Cell* **19**: 247-258.

Zhang, J., Mahdi, A.A., Briggs, G.S. and Lloyd, R.G. (2010a) Promoting and avoiding recombination: contrasting activities of the *Escherichia coli* RuvABC Holliday junction resolvase and RecG DNA translocase. *Genetics* **185**: 23-37.

Zhang, J., Mahdi, A.A., Briggs, G.S. and Lloyd, R.G. (2010b) Promoting and avoiding recombination: contrasting activities of the *Escherichia coli* RuvABC Holliday junction resolvase and RecG DNA translocase. *Genetics* **185**.

TABLE S2. DNA transfer and homologous recombination in conjugational crosses and P1 transductions

Strain	Relevant genotype		Conjugational crosses: No. Expts	Conjugational crosses: Relative viability	Relative yield of transconjugants: x KL548 to Pro$^+$	Relative yield of transconjugants: x Hfr GY2200 λ plaques	Relative yield of transconjugants: x Hfr GY2200 Thr$^+$ Leu$^+$	Relative yield of transconjugants: x Hfr KL226 to Pro$^+$	P1 Transductions: No. Expts	P1 Transductions: Relative viability	P1 Transductions: Relative yield of Leu$^+$ transductants
AB1157	*ruv*$^+$ *priB*$^+$ *rec*$^+$			**1.0 =**	**1.0 =**	**1.0 =**	**1.0 =**	**1.0 =**		**1.0 =**	**1.0 =**
		Mean	43	1.57E+08	1.47E+07	4.39E+06	7.05E+06	8.75E+06	54	1.30E+09	5.20E-05
		SE		4.84E+06	6.63E+05	4.27E+05	3.22E+05	4.25E+05		3.63E+07	4.16E-06
AM2077	*ΔpriB*		4	**0.98**	**0.89**	**1.06**	**0.83**	**1.05**	3	**0.98**	**0.40**
				0.12	0.08	0.17	0.11	0.21		0.07	0.007
AM2078	*ΔpriB ΔruvABC*		9	**0.28**	**0.0017**	**0.72**	**0.026**	**0.034**	3	**0.33**	**0.011**
				0.026	0.00061	0.05	0.0036	0.0038		0.040	0.0003
AM2089	*ΔpriB ΔrecG*		4	**0.77**	**0.85**	**0.97**	**0.69**	**0.69**	3	**0.88**	**0.49**
				0.06	0.11	0.060	0.08	0.13		0.03	0.11
AM2094	*ΔruvABC recA*		3	**0.65**	**0.60**	**0.64**	**0.000006**	**0.000015**	ND		
				0.081	0.084	0.281	0.0000007	0.000005			
AM2095	*ΔruvABC recF*		4	**0.65**	**0.81**	**0.91**	**0.78**	**0.84**	3	**0.25**	**0.44**
				0.14	0.05	0.17	0.10	0.16		0.02	0.03
AM2096	*ΔpriB ΔruvABC recA*		3	**0.48**	**0.59**	**0.84**	**0.000024**	**0.000023**	ND		
				0.041	0.103	0.212	0.0000011	0.000013			
AM2097	*ΔpriB ΔruvABC recF*		6	**0.57**	**0.64**	**0.87**	**0.37**	**0.37**	3	**0.65**	**0.14**
				0.07	0.03	0.22	0.04	0.13		0.06	0.01
AM2106	*ΔpriB recF*		4	**0.84**	**0.89**	**0.61**	**0.44**	**0.40**	3	**0.24**	**0.27**
				0.07	0.08	0.13	0.0733	0.04		0.04	0.04
AM2123	*ΔrecG*		4	**0.82**	**0.70**	**0.89**	**0.35**	**0.25**	3	**0.92**	**0.14**
				0.08	0.07	0.07	0.04	0.03		0.06	0.01
AM2124	*ΔruvABC ΔrecG*		5	**0.28**	**0.21**	**0.67**	**0.0018**	**0.0014**	3	**0.1738**	**0.0011**
				0.07	0.03	0.10	0.00063	0.00025		0.01347	0.0005
AM2131	*ΔpriB ΔruvABC ΔrecR*		4	**0.67**	**0.83**	**0.84**	**0.19**	**0.15**	3	**0.82**	**0.14**
				0.08	0.12	0.11	0.047	0.019		0.072	0.006
AM2132	*ΔpriB ΔruvABC ΔrecO*		3	**0.57**	**0.82**	**0.56**	**0.18**	**0.22**	3	**0.80**	**0.13**
				0.06	0.04	0.10	0.025	0.05		0.02	0.01
AM2133	*ΔpriB ΔruvABC recJ284*		3	**0.42**	**0.42**	**0.86**	**0.088**	**0.14**	3	**0.05**	**0.11**
				0.08	0.04	0.05	0.006	0.04		0.004	0.01
AM2134	*ΔpriB ΔruvABC ΔrecQ*		3	**0.51**	**0.55**	**0.65**	**0.079**	**0.10**	3	**0.04**	**0.08**
				0.03	0.12	0.14	0.01	0.01		0.004	0.014

AM2138	*ΔpriB ΔrecR*									
		3	**1.06**	**0.90**	**1.02**	**0.55**	**0.55**	3	**0.78**	**0.20**
			0.12	0.13	0.15	0.08	0.11		0.18	0.04
AM2139	*ΔpriB Δrec0*									
		3	**0.86**	**1.13**	**0.86**	**0.51**	**0.46**	3	**0.21**	**0.24**
			0.05	0.12	0.03	0.08	0.04		0.01	0.03
AM2140	*ΔpriB recJ284*									
		3	**1.04**	**1.04**	**1.22**	**0.51**	**0.64**	3	**0.27**	**0.38**
			0.10	0.16	0.13	0.04	0.08		0.02	0.03
AM2141	*ΔpriB ΔrecQ*									
		3	**1.12**	**0.88**	**0.93**	**0.72**	**0.56**	3	**0.36**	**0.48**
			0.15	0.24	0.04	0.06	0.11		0.02	0.02
AM2142	*ΔpriB ΔrecB*									
		3	**0.25**	**0.15**	**0.56**	**0.00088**	**0.00050**	3	**0.22**	**0.0042**
			0.01	0.01	0.04	0.000014	0.000004		0.02	0.00021
AM2147	*ΔruvABC ΔrecR*									
		3	**0.85**	**0.72**	**0.77**	**0.58**	**0.45**	3	**0.53**	**0.42**
			0.05	0.13	0.02	0.13	0.11		0.10	0.03
AM2148	*ΔruvABC ΔrecO*									
		3	**0.68**	**0.65**	**0.74**	**0.51**	**0.52**	3	**0.50**	**0.47**
			0.06	0.18	0.15	0.09	0.05		0.06	0.02
AM2149	*ΔruvABC recJ284*									
		3	**0.62**	**0.82**	**1.09**	**0.49**	**0.75**	3	**0.56**	**0.29**
			0.01	0.22	0.11	0.06	0.10		0.04	0.04
AM2160	*ΔrecR*									
		3	**1.04**	**0.98**	**0.94**	**0.85**	**0.64**	3	**0.89**	**0.65**
			0.12	0.24	0.27	0.22	0.14		0.04	0.01
AM2161	*ΔrecO*									
		3	**0.94**	**0.89**	**0.87**	**0.71**	**0.66**	3	**0.97**	**0.65**
			0.05	0.18	0.02	0.04	0.10		0.07	0.01
N2446	*recJ284*									
		3	**0.92**	**0.85**	**0.96**	**0.72**	**0.72**	3	**0.98**	**0.64**
			0.12	0.22	0.07	0.01	0.07		0.13	0.01
N4361	*recF143*									
		3	**1.01**	**0.81**	**1.03**	**0.78**	**0.62**	3	**0.80**	**0.64**
			0.11	0.10	0.17	0.02	0.06		0.07	0.03
N4454	*ΔruvABC*									
		6	**0.64**	**0.77**	**0.73**	**0.45**	**0.45**	6	**0.71**	**0.18**
			0.06	0.13	0.12	0.06	0.05		0.04	0.01
N7079	*ΔrecQ*									
		3	**1.01**	**0.93**	**0.79**	**0.89**	**0.98**	3	**0.94**	**0.90**
			0.16	0.12	0.12	0.10	0.09		0.12	0.04
N7082	*ΔruvABC ΔrecQ*									
		3	**0.67**	**0.68**	**0.87**	**0.48**	**0.57**	3	**0.48**	**0.33**
			0.03	0.12	0.21	0.09	0.04		0.03	0.01
N7911	*ΔpriB dnaC809,820*									
		4	**0.89**	**1.23**	**1.43**	**1.00**	**1.17**	3	**0.73**	**0.77**
			0.08	0.07	0.20	0.10	0.07		0.11	0.04
N7915	*ΔpriB dnaC809,820 ΔruvABC*									
		4	**0.61**	**0.85**	**1.16**	**0.25**	**0.56**	3	**0.73**	**0.19**
			0.07	0.06	0.12	0.02	0.07		0.05	0.01
N7918	*ΔpriB dnaC809,820 ΔpriC*									
		5	**0.85**	**0.99**	**1.30**	**0.35**	**1.56**	3	**1.00**	**0.35**
			0.10	0.07	0.28	0.05	0.11		0.09	0.02
N7926	*ΔpriB dnaC809,820 ΔpriC ΔruvABC*									
		6	**0.20**	**0.0028**	**0.93**	**0.036**	**0.29**	3	**0.32**	**0.008**
			0.01	0.0006	0.13	0.005	0.03		0.05	0.002

N7933	*ΔpriC*	3	**1.00**	**1.00**	**0.90**	**0.89**	**0.71**	3	**1.10**	**1.06**
			0.01	0.14	0.19	0.05	0.04		0.10	0.07
N7934	*ΔruvABC ΔpriC*	3	**0.52**	**0.68**	**0.58**	**0.27**	**0.47**	2	**0.51**	**0.14**
			0.07	0.03	0.02	0.04	0.04		0.06	0.02
N7938	*ΔpriB lexA3 ΔruvABC*	3	**0.46**	**0.58**	**0.76**	**0.09**	**0.15**	3	**0.47**	**0.011**
			0.06	0.03	0.15	0.02	0.01		0.03	0.001
N7940	*ΔpriB sfiA11 ΔruvABC*	3	**0.29**	**0.0055**	**0.70**	**0.046**	**0.02**	3	**0.25**	**0.0099**
			0.03	0.00	0.02	0.01	0.00		0.02	0.0045
N7945	*ΔruvABC rus-2*	3	**0.84**	**1.02**	**1.07**	**1.04**	**1.01**	3	**0.91**	**0.77**
			0.08	0.03	0.09	0.13	0.03		0.09	0.05
N7946	*ΔruvABC rus-2 ΔpriB*	3	**0.76**	**1.07**	**1.33**	**0.56**	**0.46**	3	**1.03**	**0.23**
			0.05	0.24	0.32	0.06	0.08		0.01	0.02
N7947	*rpoB[G1260D]*	3	**1.86**	**1.64**	**0.69**	**0.75**	**0.84**	3	**1.96**	**1.06**
			0.19	0.25	0.09	0.10	0.08		0.18	0.09
N7948	*rpoB[G1260D] ΔruvABC*	3	**1.41**	**1.06**	**0.47**	**0.26**	**0.28**	3	**1.53**	**0.24**
			0.16	0.18	0.12	0.02	0.02		0.20	0.06
N7959	*ΔruvABC rus-1*	3	**0.93**	**1.18**	**1.42**	**1.04**	**1.10**	3	**0.87**	**1.00**
			0.07	0.21	0.20	0.15	0.18		0.17	0.14
N7962	*rpsF292*	3	**0.93**	**1.18**	**1.22**	**0.99**	**1.17**	3	**0.93**	**0.82**
			0.03	0.09	0.20	0.15	0.02		0.13	0.07
N7964	*rpoB[G1260D] ΔruvABC ΔpriB*	4	**1.32**	**0.70**	**0.72**	**0.17**	**0.16**	3	**0.80**	**0.13**
			0.12	0.15	0.12	0.01	0.02		0.05	0.01
N7975	*ΔruvABC rus-1 ΔpriB*	3	**0.83**	**1.19**	**1.34**	**0.85**	**0.73**	3	**0.82**	**0.45**
			0.09	0.15	0.22	0.09	0.07		0.06	0.04
N7985	*rpsF292 ΔrecG*	3	**0.78**	**1.29**	**1.27**	**0.73**	**0.91**	2	**0.96**	**0.43**
			0.10	0.16	0.22	0.09	0.04		0.06	0.03
N7986	*rpsF292 ΔruvABC*	3	**0.60**	**1.13**	**1.15**	**0.51**	**0.52**	3	**0.42**	**0.15**
			0.02	0.40	0.14	0.07	0.03		0.06	0.01
N7987	*rpsF292 ΔrecG ΔruvABC*	3	**0.23**	**0.28**	**1.06**	**0.0024**	**0.0020**	3	**0.16**	**0.0048**
			0.02	0.13	0.21	0.0007	0.0001		0.03	0.0005
N8035	*ΔpriB ΔruvABC recB*	5	**0.19**	**0.000065**	**0.92**	**0.00025**	**0.00056**	ND		
			0.03	0.000012	0.05	0.000023	0.000035			

Mating in conjugational crosses was for 30 (KL548), 40 (KL226) or 60 (GY2200) min and the transconjugant class selected is as indicated. The phage P1 donor for transductions was W3110. Values for wild type control strain AB1157 are set at 1, with the actual mean values over all experiments shown below ± standard errors (SE) as indicated. Mutant strains were tested in groups of 3-5 in parallel with AB1157 and the values shown in bold are mean yields relative to AB1157 in each of 3 or more independent experiments as indicated, with standard errors shown below the mean. ND = not determined.

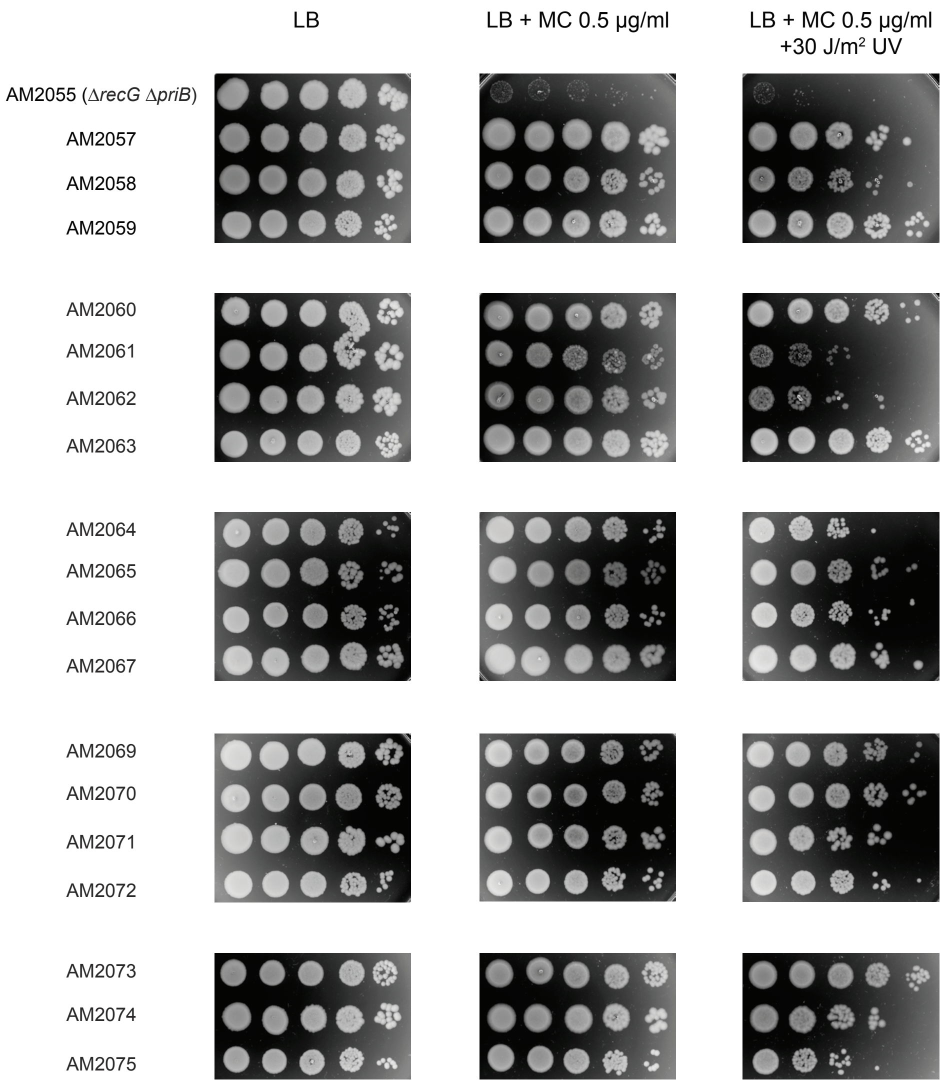

Figure. S1. Suppressors of the mitomycin C sensitive phenotype of deletion *recG priB* strain, AM2055. The suppressor strain number is shown on the left. Each strain is listed in Table S1. For each strain, cultures were grown in LB broth to an A_{650} of 0.4 and then diluted in 10-fold steps from 10^{-1} to 10^{-5}. 10 μl samples of each dilution were spotted on plates of the agar media indicated. The plates were photographed after 24 h incubation at 37C.

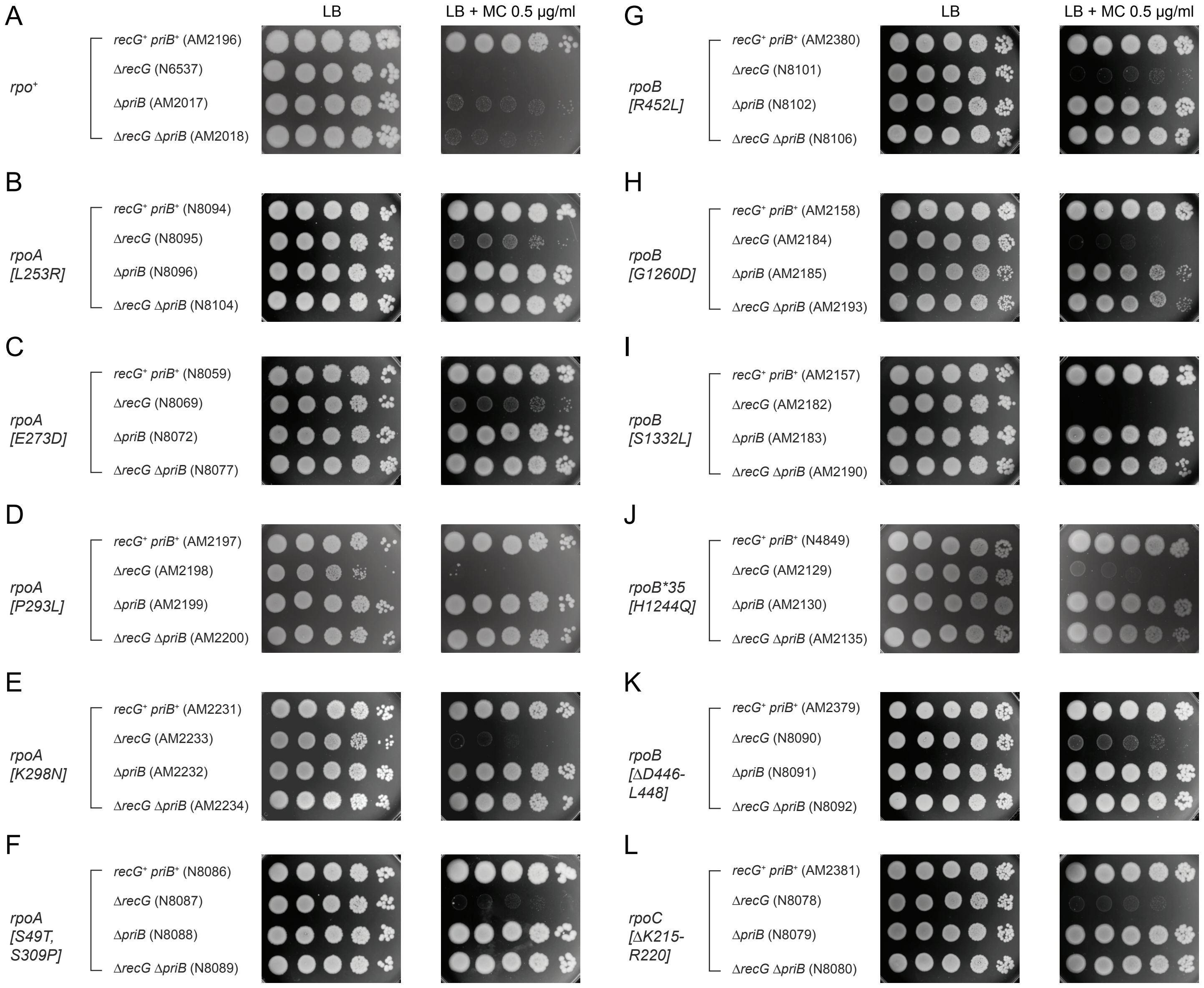

Figure. S2. Effect of mutation of RNA polymerase subunits on sensitivity to mitomycin C. Strain genotypes are identified on the left of each panel along with strain numbers in parentheses. For each strain, cultures were grown in LB broth to an A_{650} of 0.4 and then diluted in 10-fold steps from 10^{-1} to 10^{-5}. 10 μl samples of each dilution were spotted on plates of the agar media indicated. The plates were photographed after 24 h incubation at 37C.

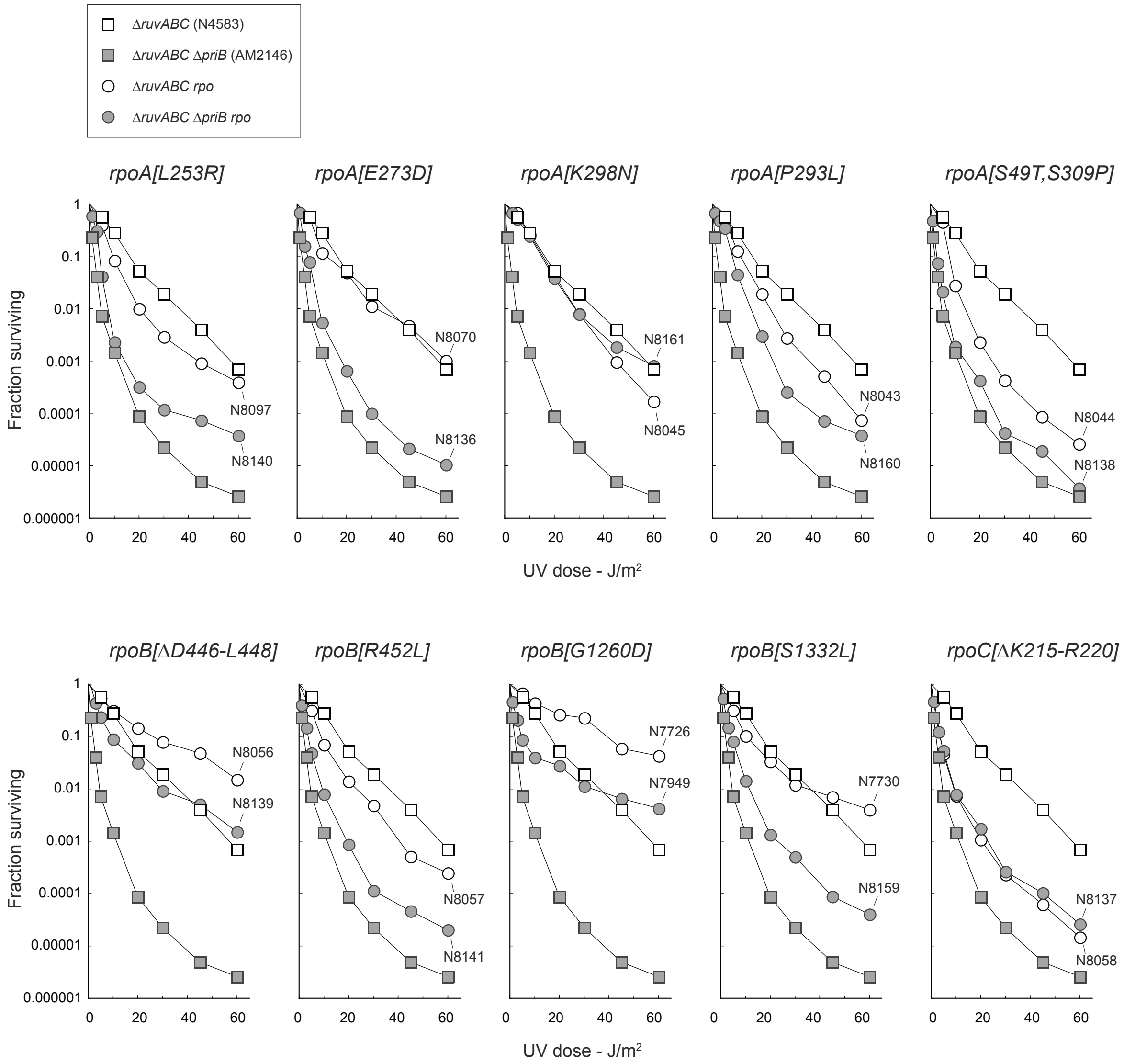

Figure. S3. Effect of mutation of RNA polymerase subunits of survival of UV-irradiated *ruvABC* and *priB ruvABC* strains. The *rpo* wild type strains N4583 and AM2146 are included in each panel for purposes of comparison.
